# Systematic review and meta-analysis of AI-based conversational agents for promoting mental health and well-being

**DOI:** 10.1038/s41746-023-00979-5

**Published:** 2023-12-19

**Authors:** Han Li, Renwen Zhang, Yi-Chieh Lee, Robert E. Kraut, David C. Mohr

**Affiliations:** 1 Department of Communications and New Media, National University of Singapore, Singapore, 117416 Singapore; 2 Department of Computer Science, National University of Singapore, Singapore, 117416 Singapore; 3 Human-Computer Interaction Institute Carnegie Mellon University, Pittsburgh, PA 15213 USA; 4 Center for Behavioral Intervention Technologies, Department of Preventive Medicine, Northwestern University, Chicago, IL 60611 USA

**Keywords:** Public health, Quality of life

## Abstract

Conversational artificial intelligence (AI), particularly AI-based conversational agents (CAs), is gaining traction in mental health care. Despite their growing usage, there is a scarcity of comprehensive evaluations of their impact on mental health and well-being. This systematic review and meta-analysis aims to fill this gap by synthesizing evidence on the effectiveness of AI-based CAs in improving mental health and factors influencing their effectiveness and user experience. Twelve databases were searched for experimental studies of AI-based CAs’ effects on mental illnesses and psychological well-being published before May 26, 2023. Out of 7834 records, 35 eligible studies were identified for systematic review, out of which 15 randomized controlled trials were included for meta-analysis. The meta-analysis revealed that AI-based CAs significantly reduce symptoms of depression (Hedge’s g 0.64 [95% CI 0.17–1.12]) and distress (Hedge’s g 0.7 [95% CI 0.18–1.22]). These effects were more pronounced in CAs that are multimodal, generative AI-based, integrated with mobile/instant messaging apps, and targeting clinical/subclinical and elderly populations. However, CA-based interventions showed no significant improvement in overall psychological well-being (Hedge’s g 0.32 [95% CI –0.13 to 0.78]). User experience with AI-based CAs was largely shaped by the quality of human-AI therapeutic relationships, content engagement, and effective communication. These findings underscore the potential of AI-based CAs in addressing mental health issues. Future research should investigate the underlying mechanisms of their effectiveness, assess long-term effects across various mental health outcomes, and evaluate the safe integration of large language models (LLMs) in mental health care.

## Introduction

Conversational agents (CAs), or chatbots, have shown substantial promise in the realm of mental health care. These agents can assist with diagnosis, facilitate consultations, provide psychoeducation, and deliver treatment options^1–3^, while also playing a role in offering social support and boosting mental resilience^4–6^. Yet, a majority of these CAs currently operate on rule-based systems, which rely on predefined scripts or decision trees to interact with users^7^. While effective to a certain degree, these rule-based CAs are somewhat constrained, primarily due to their limited capability to understand user context and intention. Recent advancements in artificial intelligence (AI), such as natural language processing (NLP) and generative AI, have opened up a new frontier--AI-based CAs. Powered by NLP, machine learning and deep learning, these AI-based CAs possess expanding capabilities to process more complex information and thus allow for more personalized, adaptive, and sophisticated responses to mental health needs^8,9^.

Despite their advantages, AI-based CAs carry risks, such as privacy infringement, biases, and safety issues^10^. Their unpredictable nature may generate flawed, potentially harmful outcomes leading to unexpected negative consequences^11^. To ensure the safe and effective integration of AI-based CAs into mental health care, it is imperative to comprehensively review the current research landscape on the use of AI-based CAs in mental health support and treatment. This will inform healthcare practitioners, technology designers, policymakers, and the general public about the evidence-based effectiveness of these technologies, while identifying challenges and gaps for further exploration.

A plethora of research has examined the effectiveness of CAs in influencing mental health, indicating that CAs can effectively mitigate symptoms of depression, anxiety, and distress, while also fostering well-being and quality of life^3,12–15^. However, these reviews have largely focused on specific types of CA^12^ or particular types of mental disorders^13,14^. Two comprehensive systematic reviews and meta-analyses^3,15^ provide evidence that supports the effectiveness of various types of CAs across a range of mental health outcomes. However, the over-representation of studies utilizing rule-based CAs in these reviews leaves the effectiveness of AI-based CAs in improving mental health remains underexplored. Moreover, the rapid progress in generative AI, such as Large Language Models (LLMs), necessitates an exploration of this technology’s potential and pitfalls, amidst uncertainties associated with its deployment in mental health care^16^. Yet, the latest studies on these advanced technologies have not been incorporated into review papers, and thus little is known about their effectiveness compared to other types of AI-based CAs for mental health support. Beyond clinical effectiveness, user experience is vital in impacting clinical outcomes. Nonetheless, prior reviews have not conclusively addressed user experience with AI-based CAs in mental health care or elucidated the factors driving the success of AI-based CA interventions.

This systematic review and meta-analysis aims to evaluate the effects of AI-based CAs on psychological distress and well-being, and to pinpoint factors influencing the effectiveness of AI-based CAs in improving mental health. Specifically, we focus on experimental studies where an AI-based CA is a primary intervention affecting mental health outcomes. Additionally, we conduct narrative synthesis to delve into factors shaping user experiences with these AI-based CAs. To the best of our knowledge, this review is the most up-to-date synthesis of evidence regarding the effectiveness of AI-based CAs on mental health. Our findings provide valuable insights into the effectiveness of AI-based CAs across various mental health outcomes, populations, and CA types, guiding their safe, effective, and user-centered integration into mental health care.

## Results

### Results of systematic review

**Fig. 1:**
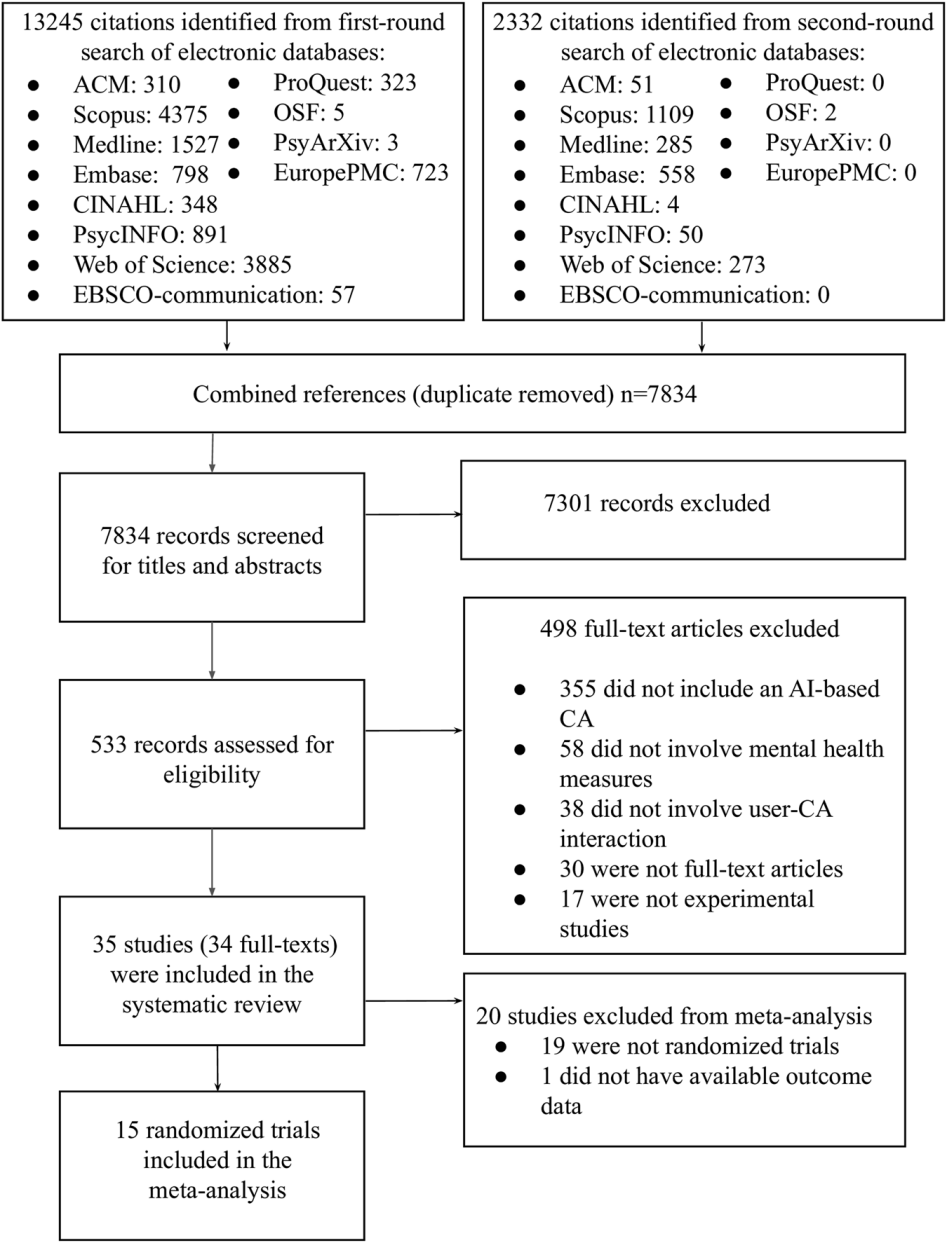
PRISMA flow diagram. Search and study selection process.

Searches of twelve databases identified 7834 unique citations (Fig. 1). We excluded 7301 records based on titles and abstracts, resulting in 533 records for full-text review. A total of 35 studies from 34 full-text articles met the inclusion criteria and were included in the systematic review for narrative synthesis. Among the 35 studies, one randomized trial^17^ did not report sufficient data for calculating pooled effect size and 19 studies were not randomized trials, leaving 15 randomized trials eligible for meta-analysis to estimate the effectiveness of AI-based CAs on psychological outcomes. Table 1 presents selected major characteristics of studies included in the systematic review (additional details are presented in Supplementary Table 1 and Supplementary Table 2).

**Table 1:** Major characteristics of studies included in the systematic review.

Study and sample characteristics				Intervention characteristics			CA design characteristics			Mechanisms	Outcomes	Note
Author, year, region	Study type, duration	Population type	Sample size	Target condition	Deployment	CA name, role	Response generation approach, AI framework/ technique	Interaction mode	Delivery platform	Therapeutic approach	Psychological outcomes (and measures)	Included in meta-analysis
Prochaska et al. (2021)^25^; USA	RCT [8 weeks]	Subclinical [adults screened with SUDs]	180	Substance use disorders	Stand-alone	Woebot-SUDs; psychotherapy/education	Retrieval-based [NLP]	Text-based	Smartphone app	Integrative approach [DBT, CBT, mindfulness]	Depression, anxiety [PHQ-8, GAD-7]	Yes
Bird et al. (2018)^48^; UK	RCT [15 min]	Nonclinical [college students]	213	Problem distress	Stand-alone	MYLO; psychotherapy	Retrieval-based [NLP]	Text-based	Web-based	MOL	Problem distress, depression, anxiety, stress [DASS-21]	Yes
Klos et al. (2021)^46^; Argentina	RCT [8 weeks]	Nonclinical [college students]	181	Depression and anxiety	Stand-alone	Tess; psychotherapy/education	Retrieval-based [NLP, emotion algorithm]	Text-based	Facebook messenger	Integrative approach [CBT, EFT, SFBT, motivational interviewing]	Depression, anxiety [PHQ-9, GAD-7]	Yes
Ogawa et al. (2022)^35^; Japan	RCT [5 months]	Clinical [older adults diagnosed with Parkinson’s disease]	20	Parkinson’s disease	Integrated with video conference	No name; teleconsultation	Retrieval-based [NLP]	Voice- based	Tablet app	NR	Depression [BDI-II]	Yes
Terblanche et al. (2022)^44^; UK	RCT [6 months]	Nonclinical [college students]	268	Psychological well-being	Stand- alone	Vici; coach for goal attainment	Retrieval-based [NLP]	Text-based	Telegram messenger	NR	Psychological well-being, perceived stress, mental resilience [WEMWBS, PSS, BRS]	Yes
Fitzpatrick et al. (2017)^26^; USA	RCT [2 weeks]	Subclinical [college students screened with depression and anxiety]	70	Depression and anxiety	Stand- alone	Woebot; psychotherapy/ education	Retrieval-based [NLP]	Text-based	Smartphone app	CBT	Depression, anxiety, positive and negative affect [PHQ-9, GAD-7, PANAS]	Yes
Romanovskyi et al. (2021)^27^; Ukraine	RCT [4 weeks]	Subclinical [college students screened with depression and/or anxiety]	82	Depression, anxiety, and negative emotions	Stand- alone	Elomia; psychotherapy/ education	Generative [GPT-2, BERT, emotion algorithm]	Text-based	Smartphone app	CBT	Depression, anxiety, positive and negative affect [PHQ-9, GAD-7, PANAS]	Yes
Drouin et al. (2022)^40^; USA	RCT [20 min]	Nonclinical [college students]	417	Psychological well-being	Stand- alone	Replika; social companion	Generative (LSTM)	Multimodal	Desktop app	NR	Positive and negative affect [PANAS]	Yes
He et al. (2022)^28^; China	RCT [1 week]	Subclinical [college students screened with depression]	148	Depression	Stand- alone	XiaoE; psychotherapy/ education	Generative [NLP, DP]	Multimodal	WeChat messenger	CBT	Depression [PHQ-9]	Yes
Papadopoulos et al. (2022)^18^; UK and Japan	RCT [2 weeks]	Nonclinical [older adults in care home]	33	Psychological well-being	Stand- alone	Pepper; social assistance	Retrieval-based [NLP]	Voice- based	robot	NR	Emotional well-being, loneliness [SF-36, ULS-8]	Yes
Bennion et al. (2020)^75^; UK	RCT [20 min]	Nonclinical [older adults]	112	Problem distress	Stand- alone	MYLO; psychotherapy	Retrieval-based [NLP]	Text-based	Web-based	MOL	Problem distress, depression, anxiety, stress [DASS-21]	Yes
Liu et al. (2022)^29^; China	RCT [16 weeks]	Subclinical [college students screened with depression]	83	Depression	Stand- alone	XiaoNan; psychotherapy/ education	Retrieval-based [NLP, ML, emotion algorithm]	Multimodal	WeChat messenger	CBT	Depression, anxiety, positive and negative affect [PHQ-9, GAD-7, PANAS]	Yes
Nicol et al. (2022)^19^; USA	RCT [4 weeks]	Clinical [adolescents diagnosed with depression and anxiety]	17	Depression	Stand- alone	Woebot; psychotherapy/ education	Retrieval-based [NLP, ML]	Text-based	Smartphone app	Integrative approach [CBT, DBT, interpersonal psychotherapy]	Depression, anxiety, mental health self-efficacy [PHQ-9, GAD-7, MHSES]	Yes
Tawfik et al. (2023)^24^; Egypt	RCT [3 months]	Clinical [women diagnosed breast cancer]	150	Breast cancer	Stand- alone	ChemoFreeBot; psychoeducation	Retrieval-based [NLP]	Text-based	Whatsapp messenger	NR	Psychological distress [MSAS-PSYCH]	Yes
Sabour et al. (2023)^41^; China	RCT [3 weeks]	Nonclinical [general population]	247	Psychological distress	Stand- alone	Emohaa-ES; social companion/ emotional support	Generative [GPT]	Text-based	WeChat messenger	NR	Depression, anxiety, positive and negative affect [PHQ-9, GAD-7, PANAS]	Yes
Fulmer et al. (2018)^17^; USA	RCT [2–4 weeks]	Nonclinical [college students]	74	Depression and anxiety	Stand- alone	Tess; psychotherapy/ education	Retrieval-based [NLP, ML]	Text-based	Common instant messengers	Integrative approach [CBT, EFT, ACT, mindfulness, self-compassion therapy, interpersonal psychotherapy]	Depression, anxiety, positive and negative affect [PHQ-9, GAD-7, PANAS]	No [insufficient data reported]
Vertsberger et al. (2022)^20^; USA	Quasi-experiment [4 months]	Nonclinical [adolescents]	10387	Psychological well-being	Stand- alone	Kai.ai; psychotherapy/ education	Retrieval-based [NLP]	Text-based	Common instant messengers	Integrative approach [ACT, mindfulness, positive psychology]	Psychological well-being [WHO-5]	No [Non-RCT]
Leo et al. (2022)^36^; USA	Quasi-experiment [2 months]	Clinical [adults diagnosed with musculoskeletal condition and screened with depression and/or anxiety]	61	Depression and anxiety	Stand- alone	Wysa; psychotherapy/ education	Retrieval-based [NLP, ML]	Text-based	Smartphone app	Integrative approach [BA, CBT, DBT, mindfulness]	Depression and anxiety [PROMIS]	No [Non-RCT]
Rathnayaka et al. (2022)^42^; USA (S1)	Quasi-experiment [8 weeks]	Nonclinical [general population]	34	Mental health problems	Stand- alone	Bunji; social companionship /remote mental health monitoring	Retrieval-based [NLP, neural network model, emotion algorithm]	Text-based	Smartphone app	BA	Mood [self-report feeling check]	No [Non-RCT]
Rathnayaka et al. (2022)^42^; USA (S2)	Quasi-experiment [8 weeks]	Nonclinical [general population]	30	Mental health problems	Stand- alone	Bunji; social companionship & remote mental health monitoring	Retrieval-based [NLP, neural network model, emotion algorithm]	Text-based	Smartphone app	BA	Mood and emotion states [self-report feeling check and emotion analysis]	No [Non-RCT]
Abdollahi et al. (2017)^30^; USA	Quasi-experiment [4–6 weeks]	Subclinical [older adults in dementia and/or depression]	6	Quality of life	Stand- alone	Ryan; social companion and assistance	Retrieval-based [NLP]	Voice-based	Robot	NR	Mood [self-report Likert and caregiver evaluation]	No [Non-RCT]
Prochaska et al. (2021)^31^; USA	Quasi-experiment [8 weeks]	Subclinical [adults screened with SUDs]	101	Substance use disorders	Stand- alone	Woebot-SUDs; psychotherapy/ education	Retrieval-based [NLP]	Text-based	Smartphone app	Integrative approach [DBT, CBT, mindfulness]	Depression, anxiety [PHQ-8, GAD-7]	No [Non-RCT]
Bassi et al. (2022)^37^; Italy	Quasi-experiment [12 days]	Clinical [adults diagnosed with diabetes mellitus]	13	Depression, anxiety, and diabetes-related distress	Stand- alone	Motibot; psychotherapy/education, counseling	Retrieval-based [NLP, NLU]	Text-based	Telegram messenger	Transtheoretical model of change	Depression, anxiety, stress, psychological well-being, diabetes-related distress [PHQ-9, GAD-7, PSS-10, WHO-5, PAID-5]	No [Non-RCT]
Goga et al. (2022)^32^; Romania	Quasi-experiment [several four-minute sessions]	Subclinical [adults screened with PTSD]	31	PTSD	Integrated with EMDR	No name; EMDR coordination	Retrieval-based [NLP, ML]	Multimodal	EMDR	NR	Psychological distress, anxiety [IES-R, STAI]	No [Non-RCT]
Tulsulkar et al. (2021)^38^; Singapore	Quasi-experiment [6 days]	Clinical [older adults diagnosed with cognitive impairments]	14	Psychological well-being	Stand- alone	Nadine; social companionship/ assistance	Retrieval-based [NLP, emotion algorithm]	Voice-based	robot	NR	Emotion states [OERS]	No [Non-RCT]
Trappey et al. (2022)^45^; Taiwan	Quasi-experiment [NR]	Nonclinical [college students]	34	Psychological well-being	Integrated with counseling system	VRECC; psychotherapy, counseling	Generative [BERT, NLU, NLG]	Multimodal	VR	Person-centered therapy	Stress, psychological sensitivity [Student Stress Survey]	No [Non-RCT]
Leo et al. (2022)^39^; USA	Quasi-experiment [2 months]	Clinical [orthopedic patients screened with depression and/or anxiety]	153	Depression and anxiety	Stand- alone	Wysa; psychotherapy/education	Retrieval-based [NLP, ML]	Text-based	Smartphone app	Integrative approach [CBT, BA, mindfulness]	Depression, anxiety [PROMIS]	No [Non-RCT]
De Nieva et al. (2020)^21^; Philippines	Quasi-experiment [2 weeks]	Nonclinical [adolescents]	25	Stress	Stand- alone	Woebot; psychotherapy/education	Retrieval-based [NLP]	Text-based	Smartphone app	CBT	Stress [PSS]	No [Non-RCT]
Gamborino et al. (2019)^22^; Taiwan	Quasi-experiment [4 days]	Nonclinical [children]	19	Psychological well-being, emotional support	Stand- alone	RoBoHoN; social companion	Retrieval-based [IRL, NLP, emotion algorithm]	Voice-based	robot	NR	Mood [facial expression and body gesture]	No [Non-RCT]
Pham et al. (2021)^43^; USA	Quasi-experiment [NR]	Nonclinical [community-dwelling older adults]	26	Psychological well-being	Stand- alone	No name; social companion/ assistance	Retrieval-based [NLP, emotion algorithm]	Voice-based	robot	NR	Loneliness, positive and negative affect, fatigue [ULS-8, PANAS, IFS]	No [Non-RCT]
Daley et al. (2020)^47^; Brazil	Quasi-experiment [1 month]	Nonclinical [general population]	3629	Depression, anxiety and stress	Stand- alone	Vitalk; psychotherapy/education	Retrieval-based [NLP, NLU]	Text-based	Common instant messengers	Integrative approach [CBT, positive psychology]	Depression, anxiety, stress [PHQ-9, GAD-7, DASS-21]	No [Non-RCT]
DEMİRCİ. (2018)^49^; Turkey	Quasi-experiment [2 weeks]	Nonclinical [college students]	16	Psychological well-being	Stand- alone	Woebot; psychotherapy/education	Retrieval-based [NLP]	Text-based	Smartphone app	CBT	Psychological well-being [FS]	No [Non-RCT]
Legaspi Jr. et al. (2022)^23^; Philippines	Quasi-experiment [1 week]	Nonclinical [adolescents]	10	Psychological well-being	Stand- alone	Wysa; psychotherapy/education	Retrieval-based [NLP, ML]	Text-based	Smartphone app	Integrative approach [positive psychology, mindfulness]	Stress, loneliness, worry [PSS, ULS-8, PSWQ]	No [Non-RCT]
Wrightson-Hester et al. (2023)^33^; Australia	Quasi-experiment [2 weeks]	Subclinical [young people experiencing depression, anxiety and/or low mood]	13	Mental health problems	Stand- alone	MYLO; psychotherapy	Retrieval-based [NLP, RL]	Text-based	Smartphone app	MOL	Depression, anxiety, psychiatric impairment, problem distress, mental health self-efficacy [PHQ-9, GAD-7, GHQ-12, PSYCHLOPS, General Self-Efficacy Scale]	No [Non-RCT]
Chiauzzi et al. (2023)^34^; USA	Quasi-experiment [8 weeks]	Subclinical [adults screened for depression or anxiety]	256	Depression and/or anxiety	Stand- alone	Woebot; psychotherapy/education	Retrieval-based [NLP]	Text-based	Smartphone app	Integrative approach [CBT, IPT, DBT]	Depression, anxiety [PHQ-8, GAD-7]	No [Non-RCT]

Abbreviations for therapeutic approaches: *ACT* Acceptance and commitment therapy, *BA* Behavioral Activation, *CBT* Cognitive Behavioral Therapy, *DBT* Dialectical Behavior Therapy, *EFT* Emotion-Focused Therapy, *MOL* Method of Levels, *SFBT* Solution-Focused Brief Therapy;

Abbreviations for outcome measures: *BDI-II* Beck Depression Inventory-II, *BRS* Brief Resilience Scale, *DASS-21* Depression Anxiety Stress Scales-21, *FS* The Flourishing Scale, *GAD-7* Generalized Anxiety Disorder-7, *GDS* Geriatric Depression Scale, *GHQ-12* General Health Questionnaire, *IES-R* Impact of Events Scale-Revised, *IFS* Iowa Fatigue Scale, *MHSES* Mental health Self-Efficacy Scale, *MSAS-PSYCH* Memorial Symptom Assessment Scale-Psychological symptom distress, *OERS* Observed Emotion Rating Scale, *PAID-5* Problem Areas in Diabetes-5, *PANAS* Positive and Negative Affect Scale, *PHQ-8* Patient Health Questionnaire-8, *PHQ-9* Patient Health Questionnaire-9, *PROMIS* Patient-Reported Outcomes Measurement Information System, *PSS* Perceived Stress Scale, *PSS-10* Perceived Stress Scale-10, *PSWQ* Penn State Worry Questionnaire, *SF-36* 36-Item Short Form Survey, *PSYCHLOPS* Psychological Outcome Profiles, *STAI* State-Trait Anxiety Inventory, *ULS-8* UCLA Loneliness-8, *WEMWBS* Warwick-Edinburgh Mental Wellbeing Scale, *WHO-5* 5-item World Health Organization Well-being Index;

Abbreviations for AI techniques: *ALML* Artificial Intelligence Markup Language, *DP* Deep Learning, *GPT* Generative Pre-training Transformer, *GPT-2* Generative Pre-training Transformer-2, *IRL* Interactive Reinforcement Learning, *LSTM* Long Short-Term Memory Networks, *ML* Machine Learning, *NLG* Natural Language Generation, *NLP* Natural Language Processing, *RL* Reinforcement Learning;

Other Abbreviations: *EMDR* Eye Movement Desensitization and Reprocessing, *NR* Not report, *PTSD* Post-Traumatic Stress Disorder, *SUDs* Substance Use Disorders.

1. We classified CA deployment into two categories: stand-alone: the CA operates independently without being part of any other system or application; integrated: the CA is incorporated into or combined with another system, therapy, or service, which means that the CA is a component of a larger therapeutic system or framework.

2. Study duration refers to the active period of the CA-based interventions, which did not include any subsequent follow-up periods.

Of the 35 studies included in our systematic review, 19 employed a quasi-experimental design, and 16 were randomized trials. The studies involved 17,123 participants from 15 countries and regions. Most were single-site studies, with 14 conducted in the United States and only one multi-site study conducted in the UK and Japan^18^. Studies were published between 2017 and 2023, with 27 published since 2020. The majority of studies (*n* = 28) had sample sizes under 200. Participants’ ages ranged from 10.7 to 92 years. Five studies^19–23^ focused on adolescents or children, while the rest included adult populations. In terms of gender, one study exclusively evaluated female populations^24^, and the rest included both genders. Half of the studies (*n* = 18) involved non-clinical populations, while 10 studies^25–34^ included participants with self-report or screened symptoms of mental illnesses, and another seven studies^19,24,35–39^ involved patients with diagnosed mental or physical issues. Study duration varied considerably, from several minutes to 6 months.

We extracted data on both the characteristics of the CA intervention and the technical design features of the CAs (see Table 2 for a summary). In total, 23 distinct CAs were evaluated across the 35 studies. Most commonly, CAs were used for the delivery of psychotherapy and/or psychoeducational content (*n* = 22). The integrative approach and CBT emerged as the most prevalent therapeutic approaches, represented in 11 and 6 studies respectively. Additionally, several CAs were designed to offer social assistance, companionship, or act as a source of emotional support for users^18,22,30,38,40–43^. There were also instances where CAs were employed for specific purposes such as coaching^37,44^, counseling^45^, remote monitoring^42^, teleconsultation^35^ or to coordinate within a larger system^32^. A significant majority of the studies (*n* = 32) featured CAs as independent, stand-alone systems.

**Table 2:** Summary of CA intervention and technical design characteristics.

CA intervention characteristics		CA design characteristics	
CA intervention	No. (prop.) of studies	CA design	No. (prop.) of studies
Deployment of CA		Response generation approach & AI techniques	
Stand-alone	32 (91.4%)	Retrieval-based • NLP • Machine learning • Emotion algorithm • NLU • Neural network • RL	30 (85.7%) 30 (85.7%) 7 (20%) 6 (17.1%) 2 (5.7%) 2 (5.7%) 2 (5.7%)
Integrated	3 (8.6%)	Generative • GPT • BERT • LSTM • DP	5 (14.3%) 2 (5.7%) 2 (5.7%) 1 (2.9%) 1 (2.9%)
Role of CA		Delivery platform	
Psychotherapy and/or psychoeducation	22 (62.9%)	Smartphone/ tablet app	16 (45.7%)
Social companionship and/or assistance	9 (25.7%)	Instant messenger platform	9 (25.7%)
Remote monitoring	2 (5.7%)	robot	5 (14.3%)
Coaching	2 (5.7%)	web-based	3 (8.6%)
Counseling	1 (2.9%)	VR platform	1 (2.9%)
Teleconsultation	1 (2.9%)	EMDR platform	1 (2.9%)
Coordination in EMDR	1 (2.9%)	Interaction mode	
		Text-based	24 (68.6%)
		Multimodal/voice-based	11 (31.4%)

Regarding the design characteristics of CAs, smartphone and tablet applications emerged as the most popular platforms for delivering CA interventions, featured in 16 studies. This was followed by widely used instant messenger platforms like Facebook messenger (*n* = 9), robots (*n* = 5), web-based platforms (*n* = 3), and two studies used VR and EMDR, respectively. The majority of the studies (*n* = 30) employed retrieval-based CAs to direct conversations through a set of established responses. In all of these retrieval-based CAs, NLP was leveraged to analyze the intent and context of user inputs and to select the appropriate responses. In some instances, this NLP capability was enhanced with machine learning (*n* = 7), emotion algorithm (*n* = 6), reinforcement learning (*n* = 2), natural language understanding (*n* = 2), or neural network techniques (*n* = 2) to improve learning and contextual understanding. Conversely, a smaller set of studies (*n* = 5) implemented generative CAs for mental health interventions, which can generate wholly original dialogs. Of these, one employed both GPT-2 and BERT^27^, the other four utilized GPT^41^, BERT^45^, LSTM^40^ and DP^28^, respectively. Regarding interaction mode, most studies used text-based CAs (*n* = 24). In eight studies^22,29,38,42,43,45,46^, CAs incorporated emotion AI, such as sentiment analysis, to understand users’ emotional states and address their in-situ needs. Other notable design features included personalization and customization (*n* = 20), regular check-ins (*n* = 10), mood tracking (*n* = 8), empathic responses (*n* = 7), multimedia (*n* = 5), and human-like character and personality (*n* = 2). Despite the growing significance of safety concerns regarding CAs in mental health, only 15 studies incorporated safety assessment or protection measures in CAs, such as access to human experts^20,23,39^, onboarding processes^19,20,25,26,31^, assessment of adverse events^25,29,31,34^ and automatic crises or harm identification^17,19,25,31,34,36,41,42,46,47^.

**Fig. 2:**
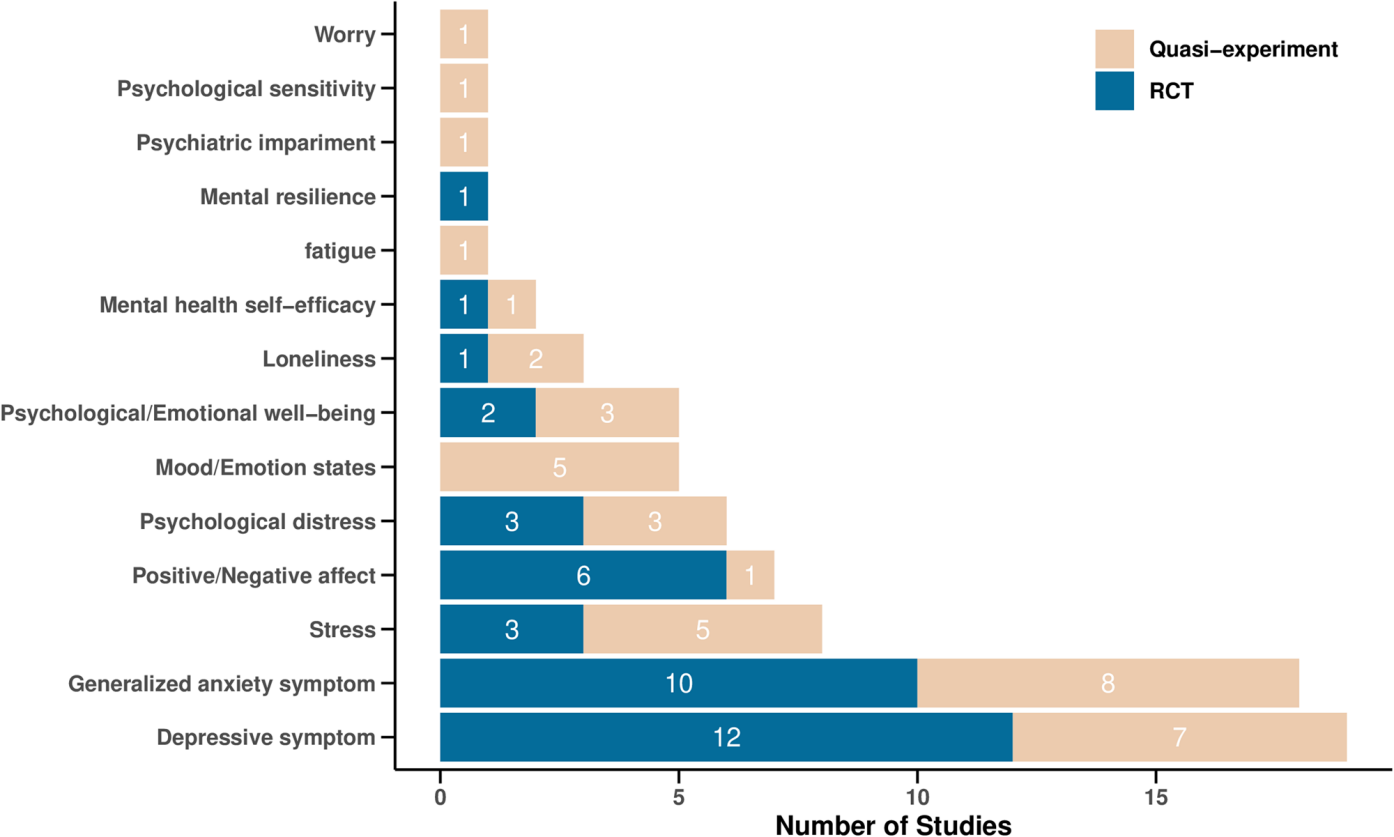
Summary of psychological outcomes evaluated in the studies. A total of 14 distinct psychological outcomes were evaluated in the 35 studies. The color of the bar denotes study type (Quasi-experiment or RCT). The number displayed on each bar represents the number of studies that evaluated the specific outcome within the given study type.

The studies evaluated a diverse range of mental health outcomes, with depression (*n* = 19) and anxiety (*n* = 18) being the most frequently assessed (see Fig. 2 for a summary). In addition to the psychotherapeutic approaches, three studies incorporated social psychological theories including empathy theory^45^, cultural competency theory^18^ and goal attainment theory^44^ to guide the CA intervention design. We did not identify any eligible studies that examined potential mediators accounting for changes in mental health outcomes, highlighting a critical gap that warrants further exploration. As for moderators, three studies probed the moderating role of user engagement. Notably, increased interactions were tied to enhanced effectiveness in reducing depression^47^ and anxiety symptoms^36,47^. However, another study^20^ observed divergent effects of interaction duration and amount on well-being, suggesting the need for more nuanced user engagement measurements to better understand the relationship between user engagement with CAs and the mental health outcomes. Of the four studies examining participant-related moderators, those with severe baseline mental health symptoms reported greater reductions in psychological distress^34,48^. Participants’ concurrent therapies or treatments, however, showed inconsistent results. Specifically, two studies noted smaller reductions in anxiety^25,34^ and depression^34^ among those engaging concurrent treatments, while another study documented larger reductions in depression in a similar cohort^31^. One study^34^ also revealed that unmarried participants experienced greater reductions in depression and anxiety than self-identified sexual minorities.

### Narrative synthesis of user engagement and experience

Of the 35 studies, 19 detailed various measures of CA engagement, including metrics such as the amount and length of conversations/messages (*n* = 13), frequency and duration of CA usage (*n* = 11), as well as the usage of specific modules or features (*n* = 5). User experiences with AI-based CAs were reported in 16 studies, primarily focusing on satisfaction (*n* = 8), acceptability (*n* = 7), and usability (*n* = 5), followed by working alliance (*n* = 4), helpfulness (*n* = 3), feasibility (*n* = 3), and likeability (*n* = 1). A total of 10 studies^17,21,26,28,29,33,37,41,46,49^ documented open-ended user feedback on their experiences interacting with AI-based CAs. Through inductive thematic analysis, these user feedbacks were classified into positive and negative experiences and further categorized into sub-themes (see Table 3). Notably, process factors fostering the formation of therapeutic relationships were frequently identified as positive experiences in eight studies, with empathic communication being the most commonly cited aspect (*n* = 5). Participants from six studies emphasized the value of specific therapeutic approaches or techniques (*n* = 3) and the richness of content (*n* = 3). Moreover, participants from three studies appraised the learning process facilitated by the CAs, and two favored accessibility. Text-based communication was regarded as a positive aspect in one study. Negative experiences predominantly revolved around communication breakdowns–when the CA failed to effectively understand, process, and respond to user input (*n* = 8). Content-related factors, both in terms of topics and formats, were indicated as unsatisfactory elements during interactions (*n* = 4). The impersonal nature of CAs was highlighted in two studies as a contributing factor to negative user experience while technical issues were reported in one study. Furthermore, in one study, participants voiced dissatisfaction regarding the CA’s lack of initiative and its interaction mode. Interestingly, one study found that participants suffering from more severe symptoms expressed a preference for human support over CAs.

**Table 3:** Narrative synthesis of open-ended user feedback.

Factors associated with positive user experience	Therapeutic alliance support (*n* = 8):
	• Empathic communication^17,26,28,29,37^ (*n* = 5)
	• Non-judgmental^33,49^ (*n* = 2)
	• Accountability (e.g., regular check-ins)^21,26^ (*n* = 2)
	• Human-like personality^26,49^ (*n* = 2)
	• Tailored feedback^28^ (*n* = 1)
	• Relationship^28^ (*n* = 1)
	Content (*n* = 6):
	• Specific therapeutic approach and techniques^21,29,37^ (*n* = 3)
	• Content richness^17,26,49^ (*n* = 3)
	Learning process^17,26,29^ (*n* = 3)
	Accessibility^17,29^ (*n* = 2)
	Interaction mode^33^ (*n* = 1)
Factors associated with negative user experience	Communication breakdowns^17,21,26,28,29,33,41,46^ (*n* = 8)
	Content (*n* = 4):
	• Topic of content^17,28,29^ (*n* = 3)
	• Format of content^17,46^ (*n* = 2)
	Impersonal^17,29^ (*n* = 2)
	Interaction mode^41^ (*n* = 1)
	Preference for human support^37^ (*n* = 1)
	Technical issues^28^ (*n* = 1)

### Results of meta-analysis

**Fig. 3:**
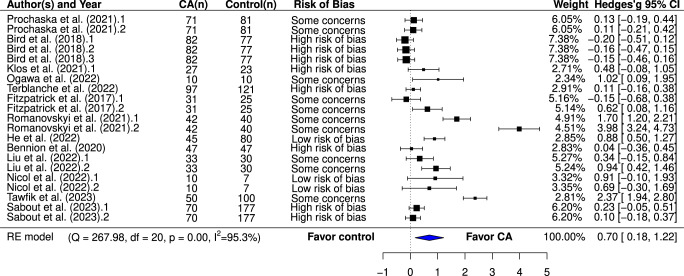
Effects of AI-based CA interventions on psychological distress. Note: the pooled effect sizes (Hedges’g) on psychological distress were reverse coded from their original values to align with the directionality of the pooled effect sizes on psychological well-being, i.e., positive effect sizes indicate a more favorable outcome for the CA intervention compared to control conditions.

**Fig. 4:**
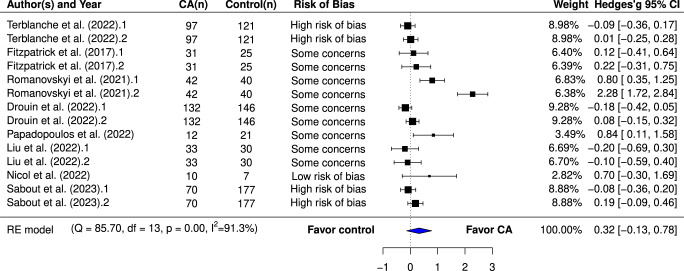
Effects of AI-based CA interventions on psychological well-being. Note: positive effect sizes indicate a more favorable outcome for the CA intervention compared to control conditions.

A total of 15 studies, involving 1744 participants, were eligible for inclusion in our meta-analysis. Among these, 13 trials examined indicators of psychological distress (Fig. 3), and eight trials assessed psychological well-being (Fig. 4). Compared to various control conditions, participants interacting with AI-based CAs exhibited a significantly greater reduction in psychological distress, with an effect size of g = 0.7 (95% CI 0.18–1.22). The “leave-one-out” sensitivity analyses demonstrated the robustness of this result, with estimated effect sizes ranging from 0.529 to 0.787. However, when we excluded two influential studies^24,27^, the overall effect sizes modestly decreased to 0.529 and 0.564, respectively but maintained the same direction and significance (refer to Supplementary Table 3). Interestingly, both of these two studies employed generative CAs, suggesting that the response generation approach of CAs could potentially influence their effectiveness. Although participants interacting with AI-based CA showed improvements in psychological well-being, this enhancement was not statistically significant (g = 0.32; 95% CI –0.13 to 0.78), perhaps because of insufficient power. Only eight trials investigated psychological well-being compared to 13 examining psychological distress. Additional meta-analyses on specific mental health outcomes, detailed in Supplementary Figs. 1–4, indicated that CA interventions significantly outperformed control conditions in ameliorating depression (g = 0.644, 95% CI 0.17–1.12). However, they did not significantly impact anxiety (g = 0.65, 95% CI –0.46 to 1.77), positive affect (g = 0.07, 95% CI –0.43 to 0.57) or negative affect (g = 0.52, 95% CI -0.67 to 1.71).

Analyses revealed significant heterogeneity for both psychological distress (Q = 267.98, *p* < 0.001, I^2^ = 95.3%) and psychological well-being (Q = 85.7, *p* < 0.001, I^2^ = 91.3%). Egger’s regression test suggested no clear publication bias (Supplementary Table 4). While AI-based CAs demonstrated high effectiveness in addressing psychological distress, we graded the quality of evidence as moderate. This decision was driven by the substantial heterogeneity observed across the studies and the wide confidence interval of the effect estimate, which cast doubts on the consistency and precision of the results. The grade of recommendation for AI-based CAs in enhancing psychological well-being was rated as low (see Supplementary Table 5 for the Summary of Findings). The overall risk of bias was low for two studies, high for five studies, and the remaining eight studies had unclear risk of bias. The most notable source of bias arose from performance bias, largely due to the lack of blinding of participants and personnel, which aligns with findings from a previous study^50^. Furthermore, the presence of attribution bias in five studies, influenced by either improper methods of addressing missing data or a significant dropout rate, might have caused deviations in the intended interventions (for a visual representation of risk of bias, see Supplementary Fig. 5).

To explore the potential sources of heterogeneity, we performed subgroup analyses focusing on various participant-, study- and CA- related moderators. Regarding psychological distress, the results showed that the ameliorative impact of CAs was more pronounced for generative CAs (g = 1.244) in contrast to retrieval-based ones (g = 0.523, F(2, 19) = 4.883, *p* = 0.019), and stronger for multimodal/voice-based agents (g = 0.828) compared to text-based ones (g = 0.665, F (2, 19) = 3.655, *p* = 0.045). Additionally, the effect was stronger when the intervention was delivered through smartphone or tablet apps (g = 0.963) and instant messengers (g = 0.751), than that observed for web-based platforms (g = –0.075, F (3,18) = 3.261, *p* = 0.046). As for participants’ characteristics, a larger effect size was observed in middle-aged/older adults (g = 0.846) in comparison with adolescents/young adults (g = 0.64, F (2, 19) = 3.691, *p* = 0.044). Moreover, the reduction in psychological distress was more pronounced in the clinical/subclinical population (g = 1.069) compared to non-clinical population (g = 0.107, F (2,19) = 7.152, *p* = 0.005). Female percentage in the sample did not moderate the effects of CA on psychological distress (g = –0.47, F (1, 19) = 0.105, *p* = 0.749). Similarly, CA intervention effects on psychological distress did not differ by the type of control groups (F (5, 20) = 2.598, *p* = 0.06). Yet, the effects of AI-based CA on psychological well-being did not exhibit significant variations associated with participants’ age (F(2,12) = 1.444, *p* = 0.274), gender (F(1, 12) = 0.462, *p* = 0.51), health status (F(2, 12) = 1.624, *p* = 0.238), the response generation approach (F(2, 12) = 1.253, *p* = 0.32), interaction mode (F(2, 12) = 1.338, *p* = 0.299) and delivery platform (F(3, 11) = 1.677, *p* = 0.23) of the CAs, or the type of control groups (F(4,14) = 0.175, *p* = 0.948). The detailed results of subgroup analysis are presented in Supplementary Table 6.

## Discussion

In this systematic review and meta-analysis, we synthesized evidence on the effectiveness and user evaluation of AI-based CAs in mental health care. Our findings suggest that these CAs can effectively alleviate psychological distress, with the most pronounced effects seen in studies employing generative AI, using multimodal or voice-based CAs, or delivering interventions via mobile applications and instant messaging platforms. CA-based interventions are also more effective among clinical and subclinical groups, and elderly adults. Furthermore, AI-based CAs were generally well-received by the users; key determinants shaping user experiences included the therapeutic relationship with the CA, the quality of content delivered, and the prevention of communication breakdowns.

Notably, we observed a significant and large effect size of AI-based CAs in mitigating psychological distress (g = 0.7) compared to small-to-moderate effects (g ranging from 0.24 to 0.47) reported in a recent review that primarily included rule-based CAs^15^. This suggests that conversational agents enhanced by advanced AI and machine learning technologies outperform their rule-based counterparts in managing psychological distress. Furthermore, the notably larger effect size of generative CAs (g = 1.244) relative to retrieval-based ones (g = 0.523) suggests that the effectiveness of CA interventions may be influenced by the response generation approach employed, which determines how well these agents are capable of simulating human conversations. Given the rapid advancements in AI technologies, further investigations are warranted to explore the potential benefits and risks of generative CAs. Identifying conditions for optimal effectiveness of different response generation approaches is vital to developing evidence-based guidelines for the implementation of various conversational agents across diverse clinical contexts.

While AI-based CAs consistently reduced psychological distress, their impact on psychological well-being was less consistent, which aligns with a previous review^51^. There are two possible explanations for this result. First, fewer studies investigated psychological well-being (*n* = 8) compared to psychological distress (*n* = 13), which could potentially curtail the statistical power necessary to detect a significant pooled effect on well-being^52^. Second, measures of psychological distress tend to be more sensitive to recent experience-induced changes, while measures of psychological well-being are typically more stable over time, requiring sustained and long-term engagement. As such, future research should explore the long-term effects of AI-based CAs to evaluate their effectiveness in promoting psychological well-being and to better understand CA effectiveness across diverse mental health outcomes.

Multimodal or voice-based CAs were slightly more effective than text-based ones in mitigating psychological distress. Their integration of multiple communication modalities may enhance social presence^53^ and deepen personalization, thus fostering a more human-like experience^54,55^ and boost the therapeutic effects^56^. In addition, a CA including text and voice functionalities might support individuals with cognitive, linguistic, literacy, or motor impairments. However, a recent study found text-based chatbots were better at promoting fruits and vegetable consumption^57^. This suggests that the effectiveness of chatbot modality may vary based on context and desired outcomes, underscoring the importance of adaptable, tailored CA designs. Moreover, a significant subgroup difference in psychological distress was noted regarding CA’s delivery platform. Mobile applications and instant messaging platforms may offer advantages in terms of reach, ease of use, and convenience when juxtaposed with web-based platforms, potentially leading to enhanced outcomes.

Our analysis also revealed that AI-based CAs were more effective in clinical and subclinical populations. This result echoes previous studies suggesting that psychological interventions are more effective for people with mental or physical health conditions compared to the general population^51^ and such effect is larger when mental health symptoms are more severe^58^. However, prior research also shows that people with more severe symptoms showed a preference for human support^37^. This underscores the need for research to untangle the complex interplay between symptom severity, CA intervention, human support, and clinical outcomes, and to pinpoint the conditions under which CAs are most effective and when human support is indispensable. Another interesting finding was that middle-aged and older adults seemed to benefit more from AI-based CAs than younger populations. One possible explanation might be the variations in engagement levels, but due to the high heterogeneity across studies, we were unable to validate these assumptions. Future research is warranted, as a prior review suggests a curvilinear relationship between age and treatment effects^59^. Notably, we did not find a significant moderating effect of gender, consistent with earlier findings demonstrating that digital mental health interventions are similarly effective across genders^60^.

In terms of user evaluation, most studies included in our review reported positive feedback for AI-based CAs, suggesting their feasibility across diverse demographic groups. Our analysis of open-ended user feedback revealed that factors such as the therapeutic relationship, content quality, and communication breakdowns were key determinants of user experience, which corresponds to previous psychotherapy research that identifies these common elements (e.g., therapeutic alliance, empathy, and therapist effect) as active ingredients contributing to therapeutic changes across various therapeutic frameworks^61^. Communication breakdowns with CAs can lead to negative user experiences, making the intervention less likely to succeed. Although retrieval-based CAs understand user context better than rule-based CAs, their limitations in generating responses can cause unnatural or repetitive interactions, potentially reducing clinical effectiveness. Despite these factors being identified as important based on qualitative user feedback, none of the included studies empirically examined their mediating or moderating effects. Future research should delve into these elements to understand the mechanisms of change and key components for successful CA interventions.

This review has its limitations. First, our broad search strategy, while exhaustive, led to considerable heterogeneity in outcome measures and results, making definitive conclusions and direct comparisons challenging. Standardized evaluation methods for clinical and non-clinical outcomes in future studies would help address this issue. Second, due to a limited number of studies reporting follow-up effects (*n* = 6) and the substantial variation in follow-up durations, we were unable to conduct a meta-analysis of the long-term effects of CA interventions on psychological outcomes. Therefore, the lasting effects of CA interventions remain unclear. Third, by only including English-language publications, we may have overlooked relevant studies in other languages, potentially limiting the generalizability of our findings. Fourth, the narrative synthesis of user experiences heavily depends on the interpretative reliability of the original studies, which may have methodological issues influencing their results. Lastly, the realm of generative AI and LLMs is evolving at an unprecedented pace. While we identified five studies using generative CAs powered by various generative AI models and frameworks, we were unable to examine the effect of specific AI models on outcomes due to the limited sample size. As the adoption of generative AI for mental health care expands, future research may benefit from differentiating the impacts of various generative AI forms.

AI-based CAs are surfacing as an impactful component in mental health care. This review provides preliminary and most up-to-date evidence supporting their effectiveness in alleviating psychological distress, while also highlighting key factors influencing effectiveness and user experience. While AI-based CAs are not designed to replace professional mental health services, our review suggests their potential to serve as a readily accessible and effective solution to address the expanding treatment gap. Future research endeavors need to delve deeper into the mechanisms and empirically evaluate the key determinants of successful AI-based CA interventions, spanning diverse mental health outcomes and populations.

## Methods

### Search strategy and selection criteria

We conducted a systematic search across twelve datasets, using a wide array of search terms. The search covered all data from the inception of each database up until Aug 16, 2022 and was later updated to include new entries up to May 26, 2023. We fine-tuned our search strategy based on previous systematic reviews^3,51,62^ to locate sources related to AI-based CAs for addressing mental health problems or promoting mental well-being. The search was limited to English-language publications. Complete lists of datasets and search strategies are detailed in Supplementary Table 7.

After removing duplicates, we screened all retrieved citations and abstracts in two stages: title/abstract screening and full-text review. Two reviewers independently reviewed all titles and abstracts for eligibility, followed by a full-text review. At each screening stage, a 10% subset of records was jointly reviewed to evaluate inter-rater reliability; disagreements were resolved through discussion, with the involvement of a third reviewer if needed. Inter-rater reliability, assessed using Cohen’s Kappa^63^, indicated near-perfect agreement for title/abstract screening (0.9) and full-text review (0.83).

The full description and examples of eligibility criteria are outlined in Supplementary Table 8. Briefly, we developed our eligibility criteria based on PICOS framework: (1) Population: all demographics or groups were eligible; (2) Intervention: we included studies that used an AI-based CA as the primary intervention, which entails a two-way interaction between a user and the CA. These AI-based CAs are defined as software agents or bots that leverage NLP, machine learning or other AI models and techniques to simulate human-like conversations. Unlike rule-based systems that depend on predefined rules or decision trees to formulate responses^7^, these agents possess the capability to understand user intent, analyze contexts, and retrieve or generate appropriate response based on the users’ input and the context of the conversation; (3) Comparator: we included studies with any comparison, ranging from active CA or human control groups to usual care, or those without a direct comparator, such as single group pre-post studies; (4) Outcome: we considered any outcomes related to psychological distress or well-being as eligible. These could be measured through self-reported questionnaires, objective metrics (e.g., audio or visual signals from passive sensing systems) or third-party evaluations; (5) Study: we included any experimental study design.

### Data management and extraction

We developed a comprehensive data extraction form and pilot-tested it on a subset of included studies to ensure reliability and reproducibility. The following data were then extracted from all included studies: publication details (author, title, journal, year), study details (region, duration, method), participant characteristics (population type, sample size, demographics), CA intervention characteristics (deployment, session, role, target condition, safety measures), CA design features (name, delivery platform, AI model/framework/technique, interaction mode, and other reported design features), therapeutic orientation (e.g., cognitive behavioral therapy; CBT), user evaluation approach (user engagement, user experience, and other reported user feedback), psychological outcomes and measures, and mechanisms (theory, moderator, mediator).

We also extracted and narratively synthesized data related to engagement and user experience of AI-based CAs from studies reporting relevant information, encompassing users’ involvement, interactions with CA interventions, and their affective and cognitive evaluations^64^. Moreover, we observed that some studies reported open-ended user feedback on their experiences with CAs, potentially providing insights into factors affecting the success of CA interventions. To analyze user feedback, two coders performed an inductive thematic analysis to identify prevalent themes in user feedback and summarized these themes narratively.

### Meta-analysis methods

To assess the effectiveness of AI-based CA interventions, we conducted a meta-analysis on randomized trials wherein participants were randomly assigned to an experimental group receiving a target CA intervention or to control groups receiving alternative treatments, information, or being placed on a waitlist. Since all of the included randomized controlled trials (RCTs) reported at least one indicator of psychological distress (i.e., distress, depression, anxiety and stress)^65^ and/or psychological well-being (i.e., psychological well-being, positive and negative affect, mental resilience, mental health self-efficacy), we performed two separate meta-analyses to estimate pooled effect sizes for these two overall psychological outcomes. Furthermore, we conducted meta-analyses for specific psychological outcomes reported by at least three trials, including depressive symptom, generalized anxiety symptom, and positive affect and negative affect.

The meta-analyses were conducted using R software (version 3.6.2) and the *metafor* package. Data were extracted from RCTs to calculate pooled effect sizes of Hedges’ g, with corresponding 95% confidence intervals and *P*-values. Hedges’ g of 0.2 indicated a small effect, 0.5 a moderate effect and 0.8 a large effect^66^. Since we expected considerable heterogeneity among RCTs, random-effects models were used for all meta-analyses a priori. Heterogeneity among trials was assessed using Cochran’s Q test and I^2^. Egger’s regression test was used to evaluate publication bias. As most trials contributed more than one observed effect size in assessing the two overall psychological outcomes, we fit two three-level random-effects meta-analytical models to account for dependencies between effect sizes, which allow effect sizes to vary between participants, outcomes, and studies^67^. We calculated Hedges’g using post-intervention outcome data that provided means and standard deviations (SDs). When SDs were not reported, they were obtained by mathematical transformation^68^. When both intention-to-treat and completer analyses were reported, we extracted and analyzed the former. For studies with multi-arm designs that included multiple experimental or control groups, we combined the means and SDs from the different arms to create a single pair-wise comparison, as suggested by the Cochrane guidelines for integrating multiple groups from a single study^69^. If a study did not report sufficient data (mean, SD, SE, 95% CI) to calculate Hedges’g, we contacted corresponding authors for missing data; studies lacking necessary data were excluded from the meta-analysis. For sensitivity analysis, we employed a “leave-one-out” method^70^ to identify influential studies and assess the robustness of estimates.

To investigate potential sources of heterogeneity, we conducted a series of subgroup analyses on the two primary psychological outcomes. In accordance with previous research, we examined participant-specific characteristics (i.e., gender, age, and health status) as well as the type of control groups^60^. Additionally, we considered three CA technical features (i.e., response generation approach, interaction mode and delivery platform) as potential moderators. We defined response generation approach as the technique a CA employs to formulate responses to user inputs. For building AI-based CAs, there are two major response generation approaches: the retrieval-based approach and the generative approach. The key distinction between the two approaches stems from their underlying mechanisms in response generation. Retrieval-based CAs, like Woebot and Wysa, rely on dialog management frameworks to track the flow of conversation and select appropriate responses from a pre-established repository of conversational utterances. In contrast, generative CAs, such as ChatGPT and Replika, leverage machine learning algorithms to learn and auto-generate responses based on a large amount of training data^71^. In terms of the interaction mode, we categorized the CAs into text-based, where users communicate with the CA via textual messages, and multimodal/voice-based, allowing users to engage with the CA using either text or vocal inputs. Furthermore, based on the medium through which the CA interacts with the users, the CAs were grouped into smartphone/tablet app, instant messaging platform, web-based platform, or robot.For categorical variables such as response generation approach, we used mixed-effects models for the subgroup analyses, while a meta-regression approach was employed for the continuous variable (i.e., gender). A detailed description of the moderators is outlined in Table 4.

**Table 4:** Description of potential moderators.

Moderator Type	Subgroup (n)	Description
Participant characteristics	Gender	Percent of female in the sample.
	Age group: • Adolescents/young adults (*n* = 10) • Middle-aged/older adults (*n* = 5)	Studies were categorized into two broad age groups based on the mean age of participants in the sample^76^: • Adolescents/young adults (13-40 years); • middle-aged/older adults (>= 40 years).
	Health status: • Clinical/subclinical population (*n* = 8) • Non-clinical population (*n* = 7)	We defined clinical population as patients with a formal diagnosis of either physical or mental issues; Subclinical population includes those screened for or self-identified as having symptoms of mental disorders, such as depression and anxiety during the study; Non-clinical consists of participants without self-identified or screened mental illness symptoms, or any diagnosed health issues. For the purposes of data analysis, we further classified health statuses into two categories: the clinical/subclinical population and the non-clinical population.
CA features	Response generation approach: • Retrieval-based (*n* = 11) • Generative (*n* = 4)	Response generation approach pertains to the technique a CA employs to formulate responses to user inputs • Retrieval-based CAs select appropriate responses from a repository of pre-existing conversational utterances; • Generative CAs automatically generate responses via machine learning algorithms.
	Interaction mode: • Text-based (*n* = 10) • Multimodal/voice-based (*n* = 5)	Text-based: users interact with the CA through textual messages; Multimodal/voice-based: users interact with the CA using either text or voice.
	Delivery platform: • Smartphone/tablet application (*n* = 6) • Instant messaging platform (*n* = 6) • Web-based platform (*n* = 2) • Robot (*n* = 1)	Delivery platform refers to the specific medium or channel through which the CA interacts with users or delivers its services. • Smartphone/tablet application: a CA deployed as a standalone application on a smartphone, tablet, or other mobile devices. • Instant messenger: a CA that operates within common instant messaging platforms, such as WhatsApp, Facebook Messenger. • Web-based platform: a CA accessible through a web browser on a computer or mobile device. • Robot: a CA integrated into a physical robot.
Study design	Control group type: • Machine control (*n* = 5) • Human control (*n* = 3) • Psychoeducation (*n* = 6) • Usual care (*n* = 2) • Waitlist (*n* = 3)	We categorized the types of control groups into five types: • Machine control: use of another type of CAs (e.g., rule-based); • Human control: human-led interventions; • Psychoeducation: information-only controls that deliver minimal psychoeducational content, such as self-help guides or basic therapeutic advice; • Usual care: standard or conventional care practices; • Waitlist: waitlist

Given that some RCTs employed a three-arm design that included two control groups, the total count of control groups surpasses the number of RCTs.

We employed the Cochrane risk of bias assessment^72^ to assess the risk of bias in the included RCTs. This assessment tool evaluates seven domains of potential bias: selection bias, performance bias, detection bias, attrition bias, reporting bias and other bias. For each domain, a trial can be categorized as having a low, high or unclear risk of bias. For the overall risk-of-bias judgment, we adopted the approach from He et al.^15^ Specifically, a trial was deemed to have a low risk of bias only if all domains were rated as low-risk. Conversely, any trial was judged to have a high risk of bias if it scored high in any domain, with the exception of performance bias. We excluded performance bias from this criterion due to the practical challenges associated with blinding participants and personnel in CA-based interventions^50^. Trials with at least one domain rated as unclear, but no domains rated as high risk were classified as having “some concerns”. For visualization, the risk of bias was represented using Review Manager (version 5.4).

To evaluate the quality of evidence presented in the two primary meta-analyses of RCTs, we used the GRADE approach^73^, which provides a holistic assessment of the combined evidence from meta-analyses. It incorporates five key considerations, and the quality of evidence may be downgraded if any of these are not adequately met. Specifically, the five considerations focus on study limitations (i.e., concerns about the risk of bias), inconsistency of the effects (i.e., variability in the effect estimates, often indicated by heterogeneity), indirectness (i.e., differences in the population, intervention, or outcome from what was intended), imprecision (i.e., uncertainty in the effect estimate, e.g., wide confidence interval), and publication bias (potential underreporting of studies with negative or null results). Conversely, factors like a large magnitude of effect or evidence of a dose-response gradient can lead to upgrades. The overall quality of evidence can be classified as high, moderate, low, or very low. The GRADE assessment is presented in the Summary of Findings table.

The study protocol was registered in PROSPERO, CRD 42023392187, and adhered to the Preferred Reporting Items for Systematic reviews and Meta-Analyses^74^ (Supplementary Table 9).

### Reporting summary

Further information on research design is available in the Nature Research Reporting Summary linked to this article.

### Supplementary information

Supplemental material

Reporting Summary

## Data Availability

Data collected and used in this meta-analysis can be requested from the corresponding author.
