# Supplementary material for: Systematic review and meta-analysis of AI-based conversational agents for promoting mental health and well-being
Source: NPJ Digit Med. 2023 Dec 19;6:236. doi: 10.1038/s41746-023-00979-5 (PMC10730549; doi:10.1038/s41746-023-00979-5)
Supplement: Supplementary file 1 — Supplemental material [file 41746_2023_979_MOESM1_ESM.pdf]

Supplementary Information

Systematic review and meta-analysis of AI-based conversational agents for promoting mental health and well-being

Han Li, PhD [1]
Renwen Zhang, PhD [1]
Yi-Chieh Lee, PhD [2]
Robert E. Kraut, PhD [3]
David C. Mohr, PhD [4]

[1] Department of Communications and New Media
National University of Singapore
Singapore 117416
Singapore

[2] Department of Computer Science
National University of Singapore
Singapore 117416
Singapore

[3] Human-Computer Interaction Institute
Carnegie Mellon University
Pittsburgh PA 15213
USA

[4] Center for Behavioral Intervention Technologies, Department of Preventive Medicine
Northwestern University
Chicago IL 60611
USA

Corresponding author:
Renwen Zhang

Email: r.zhang@nus.edu.sg
Phone: (+65) 83762508

Table of Contents

Supplementary Table 1 Additional Characteristics of included studies ... 3
Supplementary Table 2 Summary of user engagement and experiences ... 13
Supplementary Table 3 Results of sensitivity analysis ... 18
Supplementary Table 4 Assessment of publication bias using Egger's test of intercept ... 19
Supplementary Table 5 Summary of Findings and GRADE recommendations ... 20
Supplementary Table 6 Subgroup analyses for psychological distress and well-being ... 21
Supplementary Table 7 Search strings and databases ... 24
Supplementary Table 8 Eligibility criteria for study inclusion ... 31
Supplementary Table 9 PRISMA Checklists ... 33
Supplementary Fig 1. Effects of CA interventions on depressive symptoms ... 36
Supplementary Fig 2. Effects of CA interventions on generalized anxiety symptoms ... 36
Supplementary Fig 3. Effects of CA interventions on positive affect ... 37
Supplementary Fig 4. Effects of CA interventions on negative affect ... 37
Supplementary Fig 5. Risk of bias of RCTs ... 38
References ... 39

Supplementary Table 1 Additional Characteristics of included studies

	Study design	Participant demographics (age, female percent)	Other CA design features	License type	Safety measures	CA session	Theory	Moderator	Findings
Prochaska et al. (2021)[1]; USA	RCT; Exp: CA Control: waitlist	mean age=40 (12), female=65%	daily check-ins, weekly report, tailored tool or lesson	Proprietary system	crisis detection, onboarding, adverse event assessment	daily check-ins, weekly track for 8 weeks	NR	concurrent treatment	No significant change in depression (PHQ-8) and anxiety (GAD-7) by group; SUD treatment engagement was associated with less reductions in anxiety (p<0.001)
Bird et al. (2018)[2]; UK	RCT; Exp: CA Control: machine control	mean age=22.1 (7.2), female=81.6%	tailored response	NR	NR	one session with a suggested minimum of 15 minutes	NR	symptom severity	Improvements in problem distress, depression and anxiety, stress (DASS-21) in both groups with no between-group differences; Participants with moderate symptom severity experienced greater improvements in depression and anxiety than those who did not (p<0.01)
Klos et al. (2021)[3]; Argentina	RCT; Exp: CA Control: psychoeducation	age range=18-33, female=87.2%	tailored tool or lesson, empathetic response	Proprietary system	crisis detection	The chatbot initiates contact daily once a day during the initial weeks and every other day in the following weeks (8-week in total)	NR	NR	No between-group differences in depression (PHQ-9; p=0.5) and anxiety (GAD-7; p=0.9); significant decrease in anxiety in CA group (p=0.04) but not control group (p=0.33)
Ogawa et al. (2022)[4]; Japan	RCT; Exp: CA + human	mean age=66 (8.7), female=45%	NR	Proprietary platform (IBM	NR	Daily sessions for 5 months; each session	NR	NR	No between-group differences in depression (BDI-II; p>0.05)

	Control: human control			Watson Assistant)		includes at least five pairs of questions and responses			
Terblanche et al. (2022)[5]; UK	RCT; Exp: CA Control: psychoe ducation	mean age=22 (5), female=56%	progress monitoring	Proprietary system	NR	Available 24/7, free to use optionally for six months; must use the AI coach at least once before completing the monthly survey.	Goal attainm ent	NR	No significant effects or changes between the two groups over time in psychological well-being (WEMWBS), perceived stress (PSS), and resilience (BRS)
Fitzpatrick et al. (2017)[6]; USA	RCT; Exp: CA Control: psychoe ducation	mean age=22.2 (2.3), female=67%	daily check-ins, weekly report, empathic response, tailored tool or lesson	Proprietary system	onboardin g	The chatbot sent one personalized message daily to initiate conversation for 2-week	NR	NR	Decreased depression (PHQ-9; p=0.01) in CA group but not the control group; Decreased anxiety (GAD-7; p=0.004) in both groups; No group effect in positive affect and negative affect (PANAS; ns)
Romanovsk yi et al. (2021)[7]; Ukraine	RCT; Exp: CA Control: psychoe ducation	mean age=20.9 (1), female=48%	NR	Open-source model: GPT-2, BERT	NR	Unlimited access to the chatbot for 4 weeks; instruct the participants to use at any time of the day as they need	NR	NR	Decreased anxiety (GAD-7), depression (PHQ-9) and negative affect (PANAS) and increased positive affect (PANAS) in the CA group (p<0.001); No significant change in control group
Drouin et al. (2022)[8]; USA	RCT; Exp: CA Control: human controls	mean age=19.8 (3.4), female=71%	NR	Proprietary system	NR	One 20-minute session interacting with the chatbot	NR	NR	More positive affect than negative affect (PANAS) in all conditions; no significant between-group differences in positive emotions, FTF chat with human group reported slightly more

									negative emotions than the CA group (p<0.05)
He et al. (2022)[9]; China	RCT; Exp: CA Control 1: machine control; Control 2: psychoeducation	mean age=18.8 (0.9), female=37%	daily check-ins	Open-source framework (RASA)	NR	Participants complete a module (7 modules designed) per day in sequence for one week with a daily task reminder.	NR	NR	Greater decrease in depression (PHQ-9) in CA group compared to control 1 and control 2 groups (p<0.01) at both post-intervention and follow-up
Papadopoulos et al. (2022)[10]; UK and Japan	RCT; Exp: CA Control 1: machine control; Control 2: usual care	mean age=81.9 (9.8), female=66.7%	culturally competent, personalized conversation	Proprietary system	NR	Six sessions with the robot across two weeks at convenience times for residents. Each session lasted for up to 3 h, with participants free to use the robot as much or as little as they wished during the times.	Cultural competency theory	NR	Greater improvements in emotional well-being (SF-36) in CA group compared to control 2 group (p=0.02); Slight reduction in loneliness (ULS-8) in CA and control 1 group compared to control 2 group (ns).
Bennion et al. (2020)[11]; UK	RCT; Exp: CA Control: machine control	mean age=68.4 (6.5), female=73%	tailored response	NR	NR	One session with a maximum duration of 20 minutes	NR	NR	Decreased problem distress, depression, anxiety and stress (DASS-21) in both groups but no significant between-group differences

Liu et al. (2022)[12]; China	RCT; Exp: CA Control: psychoeducation	mean age=23.1 (1.8), female=55.4%	empathic response, mood track	Open-source framework (RASA)	adverse event assessment	Daily check-ins for 16 weeks	NR	NR	Greater decrease in depression (PHQ-9) and anxiety (GAD-7) in CA group compared to control group (p<0.05). The reduction of anxiety was significant only in the first 4 weeks. No significant between-group difference in positive and negative affect (PANAS)
Nicol et al. (2022)[13]; USA	RCT; Exp: CA; Control: waitlist	mean age=14.7 (1.7), female=88%	mood track, tailored tool or lesson, daily check-ins	Proprietary system	crisis detection, onboarding	Daily push notifications prompt users to check in for 4 weeks	NR	NR	Greater improvement in depression (PHQ-9) in CA group compared to control group (decreased by 3.3 units in CA group and 2 units in control group); No group difference in change in anxiety (GAD-7; ns); Greater improvement in mental health self-effectiveness (MHSES) in CA group compared to control group (increased by 6.3 units in CA group and 2.8 units in control group).
Tawfik et al. (2023)[14]; Egypt	RCT; Exp: CA; Control 1: human control; Control 2: usual care	mean age=45(7.4), female=100%	NR	Proprietary services (Microsoft Bot Framework, Azure Bot service)	NR	Interact at any time for 3 months	NR	NR	Greatest improvements in distressing psychological symptoms (MSAS-PSYCH) in CA group compared to control 1 and control 2 groups (p<0.001)
Sabour et al. (2023)[15]; China	RCT; Exp: CA; Control 1: machine	mean age=30.9 (7.9), female=79.9%	tailored support strategy to users' situation	Open-source model (GPT)	crisis detection	Participants were asked to converse with the chatbot at least once daily, and each session	NR	NR	Greater improvements in depression (PHQ-9, p=0.002) and negative affect (PANAS, p=0.003) in CA and control 1 group compared to control 2 group; no significant between-group differences in anxiety (GAD-7,

	control; Control 2: watilist					was required to last for 5-10 conversational turns, and encouraged to continue chatting for 3 weeks			p=0.556) and positive affect (PANAS, p=1.208)
Fulmer et al. (2018)[16]; USA	RCT; Exp 1: weekly CA; Exp 2: biweekly CA; Control: psychoe ducation	mean age=22.9 (4.1), female=70%	empathic, tailored tool or lesson	Proprietary system	crisis detection	7/24 availability for 2 or 4 weeks	NR	NR	Greater decreased depression (PHQ-9; p=0.03) in Exp 1 compared to control group; Greater decreased anxiety (GAD-7; p=0.045 and p=0.02) in Exp 1 and Exp 2 compared to control group; Significant difference in positive and negative affect (PANAS) between Exp 1 and control (p=0.03).
Vertsberger et al. (2022)[17]; USA	Quasi-experim ent	age range=14-18, female=NR	daily check-ins	Proprietary system	trained human companion , onboardin g	The chatbot initiates between one and three daily interactions with users; Users are encouraged to reach out to the chatbot whenever they need.	NR	user engagement	Increased well-being (WHO-5, p<0.001) over time; Time spent and number of messages sent were positively and negatively associated with well-being, respectively

Leo et al. (2022)[18]; USA	Quasi-experiment	median age=55 (42-64), female=87%	daily check-ins, weekly report, rewards	Proprietary system	NR	Participants were provided a 2-month subscription to the chatbot.	NR	user engagement	Greater improvements in anxiety (PROMIS, p=0.04) in high users (above median usage) than low users; No significant between-group differences in depression (PROMIS, p=0.15)
Rathnayaka et al. (2022)[19]; USA (S1)	Quasi-experiment	age=NR, female=NR	emotion detection and sentiment analysis, mood tracking and report, personalized conversation	Proprietary system	crisis detection	NR	NR	NR	Improved mood (self-report feeling check, p=0.003)
Rathnayaka et al. (2022)[19]; USA (S2)	Quasi-experiment	age=NR, female=NR	emotion detection and sentiment analysis, mood tracking and report, personalized conversation	Proprietary system	crisis detection	NR	NR	NR	Most users end up being happy (emotion analysis); mood trended upward over time (self-report feeling check)
Abdollahi et al. (2017)[20]; USA	Quasi-experiment	mean age=75 (7.2), female=83%	life-like, customized by user profile, endowed with a character and sense of humor, emotive, multimedia	NR	NR	Each participant had 24/7 access to the robot in their rooms for 4-6 weeks. The robot was customized to remind the user for their daily schedule	NR	NR	Improved mood (self-report Likert and caregiver evaluation)

Prochaska et al. (2021)[21]; USA	Quasi-experiment	mean age=36.8 (10), female=75.2%	mood track, empathic response, daily check-ins, weekly report, tailored lesson or tool	Proprietary system	crisis detection, onboarding, adverse event assessment	24/7 availability for 8 weeks; Daily push notifications prompt users to check in		concurrent treatment	Decreased depression (PHQ-8, p=0.005) and anxiety (GAD-7, p=0.001); Greater reductions of depression among participants currently in therapy (mean change -4.7) than those not (mean change -0.9, p=0.01)
Bassi et al. (2022)[22]; Italy	Quasi-experiment	mean age=30.1 (10.6), female=77%	tailored lesson, multimedia	Open-source framework (RASA)	NR	The interaction covered 12 sessions, each lasting 10-20 minutes; daily check-ins	NR	NR	No significant differences in depression (PHQ-9), anxiety (GAD-7), stress (PSS-10), well-being (WHO-5), and diabetes-related emotional distress (PAID-5); Self-reported perceived reductions in anxiety, depression, and stress in interviews
Goga et al. (2022)[23]; Romania	Quasi-experiment	mean age=26.2 (4.2), female=54.8%	coordinate between video, audio and tactile module	Open-source NLP techniques	NR	Several four-minute sessions	NR	NR	Decreased subjective distress associated with PTSD (IES-R, p<0.001) and decreased anxiety (STAI, p<0.001)
Tulsulkar et al. (2021)[24]; Singapore	Quasi-experiment	age=NR, female=NR	life-like, personalized conversation, empathic response, memory, The Internet of Things, multilingual, emotion engine, positive temperament	Proprietary system	NR	The robot was left running for three hours each morning for six days thereafter without the care staff to observe residents' initiative. During interaction sessions, residents could interact with	NR	NR	Participants mostly felt positive emotions during the interaction, with neutral and happiness dominated (OERS)

						Nadine; she initiated conversations when she observed residents being inactive/passive for a long time.			
Trappey et al. (2022)[25]; Taiwan	Quasi-experiment	mean age=22.7 (1.6), female=50%	empathic response, emotion detection, use of young female voice	Open-source model (BERT)	NR	Two sessions (one week apart)	empathy theory	NR	Decreased stress level (Student Stress Survey; p<0.001); no significant difference in psychological sensitivity (Student Stress Survey); more effective improvements in female than male
Leo et al. (2022)[26]; USA	Quasi-experiment	mean age=55 (15), female=83.7%	NR	Proprietary system	human counselor	Participants received 2 months of access to the chatbot	NR	NR	Clinically meaningful improvements in depression (PROMIS; p=0.006) and anxiety (PROMIS; p=0.002) in CA+usual care group; Meaningfully greater improvements in depression in CA+usual care group compared to usual care group (p=0.001) and no significant group difference between CA+usual care group and in-person care group
De Nieva et al. (2020)[27]; Philippines	Quasi-experiment	age range=16-18, female=36%	daily check-ins, weekly report, tailored tool or lesson, empathic response, multimedia	Proprietary system	NR	Daily check-ins for two weeks	NR	NR	Decreased stress level (PSS; average -15.28%); 28% of the participants experienced an increase in stress levels

Gamborino et al. (2019)[28]; Taiwan	Quasi-experiment	mean age=10.7 (1.1), female=31%	emotion detection, social actions, tailored behavior	NR	NR	Four sessions in four days, each lasted for 10 minutes	NR	NR	Maintain the user in a positive mood (facial expression and body gesture)
Pham et al. (2021)[29]; USA	Quasi-experiment	age range=60-92, female=65.4%	emotion detection, multimedia	Proprietary platform (Google API AI)	NR	One one-on-one session with the robot	NR		2/3 participants reported decreased loneliness (ULS-8); and increased positive affect (PANAS); 3/4 participants reported decreased negative affect (PANAS); 7/8 participants reported decreased fatigue (IFS)
Daley et al. (2020)[30]; Brazil	Quasi-experiment	age range=18-24, female=52%	mood track, multimedia	Proprietary system	crisis detection	Users engage four to five conversations per week with the chatbot, each lasting about 5 minutes for one month	NR	user engagement	Decreased anxiety (GAD-7; $p<0.001$), depression (PHQ-9; $p<0.001$) and stress (DASS-21; $p<0.001$);<br>Increased engagement was associated with improved depression and anxiety ($p<0.01$) but not stress ($p=0.072$)
DEMİRCİ. (2018)[31]; Turkey	Quasi-experiment	mean age=28.1 (3.3), female=50%	tailored tool or lesson, empathetic response, daily check-ins, weekly report	Proprietary system	NR	24/7 availability for two weeks; daily check-ins	NR	NR	Slight improvements in subjective well-being (FS; from 43.25 to 45.31, ns)
Legaspi Jr. et al. (2022)[32]; Philippines	Quasi-experiment	mean age=17.6 (0.7), female=30%	daily check-ins, mood track	Proprietary system	human therapist	Participants were instructed to engage in at least 10 minutes of daily conversation with the chatbot	NR	NR	Decreased stress (PSS; -8.3), loneliness (ULS-8; -6.1) and worry (PSWQ; -5.3)

						for one week			
Wrightson-Hester et al. (2023)[33]; Australia	Quasi-experiment	age range=16-24, female=54%	personalized response, co-design with target users	NR	NR	24/7 availability for two weeks	NR	NR	Decreased depression (-0.1; PHQ-9), anxiety (-1.4, GAD-7), psychiatric impairment (-0.8, GHQ-12), problem-related distress (-3.7, PSYCHLOPS) and increased self-efficacy (0.5, General Self-Efficacy Scale).
Chiauzzi et al. (2023)[34]; USA	Quasi-experiment	mean age=37.5 (12.9), female=75%	tailored conversations, mood track	Proprietary system	crisis detection, adverse events assessment	Participants were encouraged to engage with the chatbot for 8 weeks.	NR	Participants' demographic (e.g., sexual orientation) and clinical characteristics (e.g., concurrent treatment)	Significantly decreased depression (PHQ-8; mean change=-7.28, p<0.01) and anxiety (GAD-7; mean change=-7.45, p<0.01); Sexual minorities, single or divorced individuals and those with severe baseline symptoms experienced more improved outcomes. Participants with concurrent mental health treatment demonstrated lower decline in outcomes.

Abbreviations.
Abbreviations for outcome measures: BDI-II=Beck Depression Inventory-II, BRS=Brief Resilience Scale, DASS-21=Depression Anxiety Stress Scales-21, FS=The Flourishing Scale, GAD-7=Generalized Anxiety Disorder-7, GDS=Geriatric Depression Scale, GHQ-12=General Health Questionnaire, IES-R=Impact of Events Scale-Revised, IFS=Iowa Fatigue Scale, MHSES=Mental health Self-Efficacy Scale, MSAS-PSYCH=Memorial Symptom Assessment Scale-Psychological symptom distress, OERS=Observed Emotion Rating Scale, PAID-5=Problem Areas in Diabetes-5, PANAS=Positive and Negative Affect Scale, PHQ-8=Patient Health Questionnaire-8, PHQ-9=Patient Health Questionnaire-9, PROMIS=Patient-Reported Outcomes Measurement Information System, PSS=Perceived Stress Scale, PSS-10=Perceived Stress Scale-10, PSWQ=Penn State Worry Questionnaire, SF-36=36-Item Short Form Survey, PSYCHLOPS=Psychological Outcome Profiles, STAI=State-Trait Anxiety Inventory, ULS-8=UCLA Loneliness-8, WEMWBS=Warwick-Edinburgh Mental Wellbeing Scale, WHO-5=5-item World Health Organization Well-being Index

Other Abbreviations: Exp=Experimental group, NR=Not report, ns= not significant; SUDs=Substance Use Disorders

Supplementary Table 2 Summary of user engagement and experiences

Study	**Engagement and user experience (UX)**	**Open-ended feedback**
Prochaska et al. (2021)[1]; USA	Engagement: average 35±29 days; average 920±892 in-app text messages; UX: 96% lessons rated positively; 88% would recommend to others; High overall acceptability, feasibility (URP-I), satisfaction (CSQ-8), and working alliance (WAI-SR)	NR
Bird et al. (2018)[2]; UK	Engagement: average 13 minutes; UX: MYLO was rated as significantly more helpful than ELIZA (p=0.001)	NR
Klos et al. (2021)[3]; Argentina	Engagement: average 472±249 messages exchanged; higher number of messages exchanged associated with positive feedback (p=0.02);	64% positive user feedback; predominant dissatisfaction regarding misalignment of response
Ogawa et al. (2022)[4]; Japan	Engagement: Participants completed 58±155 chatbot sessions	NR
Terblanche et al. (2022)[5]; UK	NR	NR
Fitzpatrick et al. (2017)[6]; USA	Engagement: average 12.14±2.23 times; UX: Higher levels of satisfaction in experiment both overall (p<0.001) and with content (p=0.02); All participants in the experiment group reported learning something new.	Best experience (66% mentioned process-related: check-ins, learning, empathy, personality, conversation; 34% mentioned content-related: videos, games, suggestions, weekly graphs); Worst experience (74% mentioned process-related: unnatural, repetitive, glitches and looping; 26% mentioned content: emoticons, too long or short)
Romanovskyi et al. (2021)[7]; Ukraine	Engagement: 45% participants used 2 times per week, 22% once per week, 17% 4-5 times and 14% everyday;	NR
Drouin et al. (2022)[8];	NR	NR

USA		
He et al. (2022)[9]; China	Engagement: average 25.5± 26.5 interaction sessions per day, each lasted an average of 22.5±80 seconds;<br>UX: Participants in the experiment reported higher working alliance (WAQ; p=0.036) and better acceptability (AS; p<0.05) compared to control groups, but no significant difference in usability (UMUX-LITE; p=0.38).	Best experience (four themes: relationship, emotion, personalization, and practicability); Worst experience (two themes: content, technology). Suggestions (three themes: more fluent dialogue, more emotional response, and server upgrade).
Papadopoulos et al. (2022)[10]; UK and Japan	NR	NR
Bennion et al. (2020)[11]; UK	Engagement: average time spent in conversation 24± 16.5 minutes;<br>UX: MYLO was rated significantly more helpful than ELIZA; No significant differences in usability (SUS) between two conditions, but both were below the cut-off for an acceptable program; system usability was significantly associated with perceived helpfulness.	
Liu et al. (2022)[12]; China	UX: Participants reported a higher working alliance (WAI-SR) in the XiaoNan group compared to the bibliotherapy group; No significant differences on satisfaction (CSQ-8).	Best experience (76% related to process: accessibility, empathy, interesting, educational; 24% related to content: exploring depression, interactive CBT, choice list); worst experience (76% related to process: impersonal, unnatural, rigid, misunderstanding; 73% related to content: repetitive, too general, irrelevant, too simple)
Nicol et al. (2022)[13]; USA	Engagement: average used 6±7 days, with 55±7 mood check-ins, and 313±447 sent messages;<br>UX: All completed psychoeducational lessons were rated positively; over ⅔ reported feeling better after using the CA. High overall reported usability (SUS), feasibility (FIM), and acceptability (AIM).	NR
Tawfik et al. (2023)[14]; Egypt	UX: Majority of participants reported the CA was easy to use (94% agreed and strongly agreed) and its responses were useful, appropriate and informative (94%). Most reported the chatbot understood them well (72%) and was welcoming during the initial setup (88%). Most found the CA's personality to be realistic and engaging (72%) and it coped well with errors or mistakes (76%) (CUQ).	NR

Sabour et al. (2023)[15]; China	Engagement: on average 7±6.6 conversation sessions and 17±10.7 conversational turns; UX: most of the participants considered the CA as an appropriate chatting partner (77.4%), channel for emotional venting (64.5%), and made them feel heard (58.1%). More than half were satisfied with the interface (64.5%) and would recommend it to others (61.3%).	Problems with the CA: lack of initiative, unable to understand multimodal inputs, rigid conversations, out-of-context responses.
Fulmer et al. (2018)[16]; USA	UX: 86% overall satisfaction; 80% overall satisfaction with content;	Best experience (62% related to process: accessibility, empathy, learning; 38% related to content: applicability, topics, buttons); worst experience (76% related to process: unnatural, repetitive, impersonal, misunderstood; 24% related to content: not interactive, irrelevant, too fast, too general)
Vertsberger et al. (2022)[17]; USA	Engagement: average of 45.4±46.8 days and average of 214.3±220.2 messages	NR
Leo et al. (2022)[18]; USA	Engagement: overall engagement rate 72%; weekly engagement rate 57%; coach engagement rate 33%; module usage: mindfulness>CBT> sleep	NR
Rathnayaka et al. (2022)[19]; USA (S1)	NR	NR
Rathnayaka et al. (2022)[19]; USA (S2)	Engagement: On average scheduled three activities and engaged in a conversation seven times a week; Module usage: inspiration posts> gratitude journal	NR
Abdollahi et al. (2017)[20]; USA	Engagement: On average 198 dialogs per day; 2-hour interaction per day; UX: high overall likeability and acceptability (5-point Likert; average >3.5)	NR

Prochaska et al. (2021)[21]; USA	Engagement: On average 15.7±14.2 days, 12.1±8.3 modules, 600.7± 556.5 messages;<br>UX: 94% positive rating of psychoeducational lessons; High overall feasibility (URP-I; 28.5±5.7), acceptability (URP-I; 25.6± 7.3), satisfaction (CSQ; 23.2±5.5); working alliance (WAI-SR; 40.8±12.5)	NR
Bassi et al. (2022)[22]; Italy	UX: High overall perceived usability, focused attention, esthetic appeal, reward factor (all mean>3.5; UES); High overall positive user experience (4.04±0.22; UEQ) and low overall negative user experience (1.86±0.3; UEQ);	Positive experience with mindfulness audio tracks; perceived CA as empathetic, stimulating, motivating, and encouraging; participants with more severe symptoms reported a desire for human contact
Goga et al. (2022)[23]; Romania	NR	NR
Tulsulkar et al. (2021)[24]; Singapore	Engagement: average duration of conversation 15 minutes; Increased quality and duration of engagement over time (MPES)	NR
Trappey et al. (2022)[25]; Taiwan	NR	NR
Leo et al. (2022)[26]; USA	NR	NR
De Nieva et al. (2020)[27]; Philippines	UX: Moderate to high overall satisfaction (4-point Likert; 2.68-2.96); moderate to high evaluation on performance, humanity, affect (CUES; 2.84-2.97);<br>User feedback: (in) effective features (lessons and CBT, daily check-ins, gratitude journal); challenges (inappropriate response)	NR
Gamborino et al. (2019)[28]; Taiwan	NR	NR

Pham et al. (2021)[29]; USA	NR	NR
Daley et al. (2020)[30]; Brazil	Engagement: average message sent per day 8.17±3.67.	NR
DEMİRCİ. (2018)[31]; Turkey	NR	Positively valuated design aspects (no-judgment, personality, rich interactions and conversation)
Legaspi Jr. et al. (2022)[32]; Philippines	Engagement: feature usage (talk, journal, self-care, relaxation, reframing);<br>UX: High overall rating of features (average >3.5; 5-point Likert); High overall score on openness, helpfulness, content, user satisfaction, affect qualities (>3.5); lowest score on human-likeness (2.7)	NR
Wrightson-Hester et al. (2023)[33]; Australia	Engagement: first week: use 1-4 days and have 1-3 conversations with MYLO; most conversations lasted for 10-15 minutes (N=8); most used MYLO for 30-35 minutes (N=6); second week: use 1-7 days and have 1-5 conversations with MYLO; average conversation length ranged from 5 to 15 minutes; total time using MYLO ranged from 15 to 40 minutes (UES);<br>UX: rated the MYLO interface as acceptable (SUS); most participants were satisfied with text-based conversations; similar to other digitized mental health interventions but performs slightly worse than in-person therapy (SIS) more than 1000 words for a conversation was rated as helpful;	Positive experience: nonjudgmental, text-based communication; negative experience: repetition of questions, misunderstanding
Chiauzzi et al. (2023)[34]; USA	NR	NR

Abbreviations. URP-I=User Rating Profile-Intervention; CSQ-8=Client Satisfaction Questionnaire-8; WAI-SR=Working Alliance Inventory-Short Revised; AS=Acceptability Scale; WAQ=Working Alliance Questionnaire; UMUX-LITE=Usability Metric for User Experience-LITE; SUS=System Usability Scale; CBT=Cognitive Behavioral Therapy; FIM=Feasibility of Intervention Measure; AIM=Acceptability of Intervention Measure; UES=User Engagement Scale; UEQ=User Experience Scale; MPES=Menorah Park Engagement Scale; CUES=Chatbot User Evaluation Survey; SIS=Session Impact Scale.

Supplementary Table 3 Results of sensitivity analysis

Studies	Estimate	SE	Lower 95% CI	Upper 95% CI	p-value
**Psychological distress**					
Overall	0.7	0.25	0.176	1.22	0.011
-Prochaska et al. (2021)[1]	0.754	0.271	0.185	1.322	0.012
-Bird et al. (2018)[2]	0.787	0.265	0.228	1.345	0.009
-Klos et al. (2021)[3]	0.716	0.269	0.152	1.28	0.016
-Ogawa et al. (2022)[4]	0.679	0.266	0.122	1.236	0.02
-Terblanche et al. (2022)[5]	0.747	0.267	0.188	1.305	0.012
-Fitzpatrick et al. (2017)[6]	0.741	0.27	0.174	1.308	0.013
-Romanovskyi et al. (2021)[7]	0.529	0.199	0.11	0.948	0.016
-He et al. (2022)[9]	0.685	0.27	0.12	1.251	0.02
-Bennion et al. (2020)[11]	0.75	0.266	0.195	1.306	0.011
-Liu et al. (2022)[12]	0.706	0.274	0.13	1.282	0.019
-Nicol et al. (2022)[13]	0.692	0.272	0.121	1.263	0.02
-Tawfik et al. (2023)[14]	0.564	0.231	0.081	1.046	0.025
-Sabour et al. (2023)[15]	0.749	0.272	0.179	1.32	0.013
**Psychological well-being**					
Overall	0.323	0.211	-0.132	0.778	0.149
-Terblanche et al. (2022)[5]	0.388	0.242	-0.144	0.921	0.137
-Fitzpatrick et al. (2017)[6]	0.354	0.246	-0.187	0.894	0.178
-Romanovskyi et al. (2021)[7]	0.008	0.051	-0.103	0.12	0.871
-Liu et al. (2022)[12]	0.399	0.235	-0.118	0.916	0.117
-Nicol et al. (2022)[13]	0.296	0.226	-0.198	0.789	0.216
-Drouin et al. (2022)[8]	0.39	0.241	-0.14	0.921	0.133
-Papadopoulos et al. (2022)[10]	0.272	0.225	-0.218	0.762	0.25

-Sabour et al. (2023)[15]	0.374	0.245	-0.166	0.913	0.156

Note. We used the "leave-one-out" method to assess the robustness of the pooled effect sizes. Each study was sequentially removed, and the remaining datasets were reanalyzed to identify if a single study was responsible for the direction, significance and magnitude of the estimates.

Supplementary Table 4 Assessment of publication bias using Egger's test of intercept

Outcome	Estimate	SE	t value	df	p-value
Psychological distress	12.011	8.878	1.353	2.76	0.276
Psychological well-being	5.102	3.651	1.398	1.98	0.298
Depressive symptom	3.133	1.656	1.89	1.75	0.217
Generalized anxiety symptom	7.956	10.908	0.729	1.85	0.547
Positive affect	5.489	5.147	1.07	2.44	0.38
Negative affect	16.552	14.627	1.132	2.34	0.361

Supplementary Table 5 Summary of Findings and GRADE recommendations

Summary of findings:

AI-based chatbot intervention compared to Control conditions for psychological distress and psychological well-being

Patient or population: psychological distress and psychological well-being
Setting: Clinical and nonclinical settings
Intervention: AI-based chatbot intervention
Comparison: Control conditions

Outcomes	Anticipated absolute effects* (95% CI)		Relative effect (95% CI)	№ of participants (studies)	Certainty of the evidence (GRADE)	Comments
	**Risk with Control conditions**	**Risk with AI-based chatbot intervention**				
Overall psychological distress	-	SMD **0.7 SD higher** (0.18 higher to 1.22 higher)	-	1433 (13 RCTs)	⊕⊕⊕◯ Moderate[a,b]	AI-based chatbot probably results in a large decrease in overall psychological distress .
Overall psychological well-being	-	SMD **0.32 SD higher** (0.13 lower to 0.78 higher)	-	994 (8 RCTs)	⊕⊕◯◯ Low[b,c]	The evidence suggests that AI-based chatbot results in little to no difference in overall psychological well-being.

*__The risk in the intervention group__ (and its 95% confidence interval) is based on the assumed risk in the comparison group and the **relative effect** of the intervention (and its 95% CI).

CI: confidence interval; **SMD:** standardised mean difference

GRADE Working Group grades of evidence
High certainty: we are very confident that the true effect lies close to that of the estimate of the effect.
Moderate certainty: we are moderately confident in the effect estimate: the true effect is likely to be close to the estimate of the effect, but there is a possibility that it is substantially different.
Low certainty: our confidence in the effect estimate is limited: the true effect may be substantially different from the estimate of the effect.
Very low certainty: we have very little confidence in the effect estimate: the true effect is likely to be substantially different from the estimate of effect.

Explanations

[a] Wide 95% Confidence Interval
[b] Substantial heterogeneity of relevant importance.
[c] 95% Confidence Interval crosses null effect

Supplementary Table 6 Subgroup analyses for psychological distress and well-being

	Hedges' g (95% CI)	P-value	I^2 (%)	No. studies
**Psychological distress**				
Gender	-0.47 (-3.5, 2.56)	F (1,19) = 0.105, p = 0.749	95.56	13
Age Group	F (2,19) = 3.691, p = 0.044			13
Adolescents/young adults	0.64 (-0.01, 1.29)	0.052	95.58	9
Middle-aged/older adults	0.85 (-0.16, 1.86)	0.096		4
Health status	F (2, 19) = 7.152, p = 0.005			13
Clinical/subclinical population	1.07 (0.48, 1.66)	<0.01	94	8
Non-clinical population	0.11 (-0.63, 0.84)	0.764		5
Response generation approach	F (2, 19) = 4.882, p = 0.019		95.04	13
Retrieval-based	0.52 (-0.06, 1.11)	0.077		10
Generative	1.24 (0.2, 2.29)	0.022		3
Interaction mode	F (2, 19) = 3.655, p = 0.045			13
Text-based	0.66 (0.05,1.28)	0.036	95.59	10
Multimodal/ voice-based	0.83 (-0.34, 2)	0.156		3
Delivery platform	F (3, 18) =3.261, p=0.046			13
Smartphone/tablet app	0.96 (0.11, 1.81)	0.029	95.26	5
Instant messaging platform	0.75 (-0.03, 1.53)	0.058		6

Web-based platform	-0.075 (-1.38, 1.23)	0.905		2
Control group	F (5, 20) = 2.598, p = 0.06			13
Machine control	0.45 (-0.27, 1.18)	p=0.208		4
Human control	1.75 (0.41, 3.09)	p=0.013		2
Psychoeducation	0.64 (-0.03, 1.31)	p=0.06	94.01	6
Usual care	1.52 (-0.09, 3.13)	p=0.06		1
Waitlist	0.42 (-0.4, 1.24)	p=0.3		3
**Psychological well-being**				
Gender	-1.22 (-5.15, 2.7)	F (1, 12) = 0.462, p = 0.51	91.67	8
Age Group	F (2, 12) = 1.444, p = 0.274			8
Adolescents/ young adults	0.272 (-0.22, 0.76)	0.25	91.64	7
Middle-aged/ older adults	0.844(-0.7, 2.38)	0.256		1
Health status	F (2, 12) = 1.624, p = 0.238			8
Clinical/subclinical population	0.53 (-0.13, 1.19)	0.107	91.34	4
Non-clinical population	0.14 (-0.5, 0.77)	0.648		4
Response generation approach	F (2, 12) = 1.253, p = 0.32			8
Retrieval-based	0.22 (-0.41,0.85)	0.458	92.08	5
Generative	0.47 (-0.27, 1.21)	0.191		3
Interaction mode	F (2, 12) = 1.338, p = 0.299			8
Text-based	0.44 (-0.16, 1.04)	0.138		5

Multimodal/ voice-based	0.14 (-0.64, 0.92)	0.707	91.91	3
Delivery platform	F (3, 11) = 1.677, p = 0.229			8
Smartphone/tablet app	0.54 (-0.1, 1.18)	0.089	90.77	4
Instant messaging platform	-0.04 (-0.73, 0.64)	0.894		3
robot	0.844 (-0.65, 2.34)	0.24		1
Control group	F (5, 14) = 0.595, p = 0.705			8
Machine control	0.41 (-0.55, 1.37)	0.376	92.58	2
Human control	-0.05 (-1.41, 1.31)	0.936		1
Psychoeducation	0.35 (-0.36, 1.06)	0.304		4
Usual care	0.67 (-0.8, 2.19)	0.334		1
Waitlist	0.52 (-0.443, 1.48)	0.267		2

Note. Five of the RCTs [8,9,10,14,15] feature a three-arm design that with two control groups, causing the count of control groups to exceed the number of RCTs. In our subgroup analyses focusing on the moderating effects of control group type, we included each distinct control arm from these RCTs. This approach may introduce the possibility of "double counting". As a result, several treatment groups contribute to multiple effect sizes. This inclusion method may overemphasize the weight of these particular RCTs and possibly lead to an underestimation of the variance of pooled effect sizes. Therefore, the results of subgroup analyses for control type should be interpreted with caution, considering that the effect sizes from these studies are not entirely independent, which may influence the overall pooled estimate.

Supplementary Table 7 Search strings and databases

Date of search: 08/16/22 (first-round) AND 05/26/2023 (second-round)	
**Database**	**Search strings**
ACM Digital Library	("mental illness" OR "mental disorder*" OR "affective disorder*" OR "psychotic disorder*" OR "post \-traumatic stress disorder*" OR "PTSD" OR distress OR "depress*" OR anxiety OR bipolar OR schizophrenia OR psychosis OR "mental health" OR "mental wellness" OR wellbeing OR "well \-being" OR "SWB" OR happiness OR happy OR "positive affect*" OR "negative affect*" OR "positive emotion*" OR "negative emotion*" OR mood OR "life satisfaction" OR "satisfaction with life") AND ("robot*" OR "social bot*" OR "dialogue system*" OR "conversational agent*" OR "conversational bot*" OR "conversational system*" OR "conversational interface*" OR "chatbot*" OR "chat bot*" OR "chatterbot*" OR "chatter bot*" OR "chat \-bot*" OR "smartbot*" OR "smart bot*" OR "smart \-bot*" OR "virtual coach*" OR "virtual agent*" OR "embodied agent*" OR "relational agent*" OR "avatar*" OR "virtual character*" OR "animated character*" OR "virtual human*" OR "virtual assistant*" OR "digital assistant*" OR "counseling agent*")
Scopus	TITLE (("mental illness" OR "mental disorder*" OR "affective disorder*" OR "psychotic disorder*" OR "post-traumatic stress disorder*" OR "PTSD" OR distress OR "depress*" OR anxiety OR bipolar OR schizophrenia OR psychosis OR "mental health" OR "mental wellness" OR wellbeing OR "well-being" OR "SWB" OR happiness OR happy OR "positive affect*" OR "negative affect*" OR "positive emotion*" OR "negative emotion*" OR mood OR "life satisfaction" OR "satisfaction with life") AND ("robot*" OR "social bot*" OR "dialogue system*" OR "conversational agent*" OR "conversational bot*" OR "conversational system*" OR "conversational interface*" OR "chatbot*" OR "chat bot*" OR "chatterbot*" OR "chatter bot*" OR "chat-bot*" OR "smartbot*" OR "smart bot*" OR "smart-bot*" OR "virtual coach*" OR "virtual agent*" OR "embodied agent*" OR "relational agent*" OR "avatar*" OR "virtual character*" OR "animated character*" OR "virtual human*" OR "virtual assistant*" OR "digital assistant*" OR "counseling agent*")) OR ABS (("mental illness" OR "mental disorder*" OR "affective disorder*" OR "psychotic disorder*" OR "post-traumatic stress disorder*" OR "PTSD" OR distress OR "depress*" OR anxiety OR bipolar OR schizophrenia OR psychosis OR "mental health" OR "mental wellness" OR wellbeing OR "well-being" OR "SWB" OR happiness OR happy OR "positive affect*" OR "negative affect*" OR "positive emotion*" OR "negative emotion*" OR mood OR "life satisfaction" OR "satisfaction with life") AND ("robot*" OR "social bot*" OR "dialogue system*" OR "conversational agent*" OR "conversational bot*" OR "conversational system*" OR "conversational interface*" OR "chatbot*" OR "chat bot*" OR "chatterbot*" OR "chatter bot*" OR "chat-bot*" OR "smartbot*" OR "smart bot*" OR "smart-bot*" OR "virtual coach*" OR "virtual agent*" OR "embodied agent*" OR "relational agent*" OR "avatar*" OR "virtual character*" OR "animated character*" OR "virtual human*" OR "virtual assistant*" OR "digital assistant*"

	OR "counseling agent*")) AND (LIMIT-TO (DOCTYPE , "ar") OR LIMIT-TO (DOCTYPE , "cp")) AND (LIMIT-TO (LANGUAGE , "English"))
MEDLINE	AB (("mental illness" OR "mental disorder*" OR "affective disorder*" OR "psychotic disorder*" OR "post-traumatic stress disorder*" OR "PTSD" OR distress OR "depress*" OR anxiety OR bipolar OR schizophrenia OR psychosis OR "mental health" OR "mental wellness" OR wellbeing OR "well-being" OR "SWB" OR happiness OR happy OR "positive affect*" OR "negative affect*" OR "positive emotion*" OR "negative emotion*" OR mood OR "life satisfaction" OR "satisfaction with life") AND ("robot*" OR "social bot*" OR "dialogue system*" OR "conversational agent*" OR "conversational bot*" OR "conversational system*" OR "conversational interface*" OR "chatbot*" OR "chat bot*" OR "chatterbot*" OR "chatter bot*" OR "chat-bot*" OR "smartbot*" OR "smart bot*" OR "smart-bot*" OR "virtual coach*" OR "virtual agent*" OR "embodied agent*" OR "relational agent*" OR "avatar*" OR "virtual character*" OR "animated character*" OR "virtual human*" OR "virtual assistant*" OR "digital assistant*" OR "counseling agent*")) OR TI (("mental illness" OR "mental disorder*" OR "affective disorder*" OR "psychotic disorder*" OR "post-traumatic stress disorder*" OR "PTSD" OR distress OR "depress*" OR anxiety OR bipolar OR schizophrenia OR psychosis OR "mental health" OR "mental wellness" OR wellbeing OR "well-being" OR "SWB" OR happiness OR happy OR "positive affect*" OR "negative affect*" OR "positive emotion*" OR "negative emotion*" OR mood OR "life satisfaction" OR "satisfaction with life") AND ("robot*" OR "social bot*" OR "dialogue system*" OR "conversational agent*" OR "conversational bot*" OR "conversational system*" OR "conversational interface*" OR "chatbot*" OR "chat bot*" OR "chatterbot*" OR "chatter bot*" OR "chat-bot*" OR "smartbot*" OR "smart bot*" OR "smart-bot*" OR "virtual coach*" OR "virtual agent*" OR "embodied agent*" OR "relational agent*" OR "avatar*" OR "virtual character*" OR "animated character*" OR "virtual human*" OR "virtual assistant*" OR "digital assistant*" OR "counseling agent*"))
Ovid Embase	(("mental illness" OR "mental disorder*" OR "affective disorder*" OR "psychotic disorder*" OR "post-traumatic stress disorder*" OR "PTSD" OR distress OR "depress*" OR anxiety OR bipolar OR schizophrenia OR psychosis OR "mental health" OR "mental wellness" OR wellbeing OR "well-being" OR "SWB" OR happiness OR happy OR "positive affect*" OR "negative affect*" OR "positive emotion*" OR "negative emotion*" OR mood OR "life satisfaction" OR "satisfaction with life") AND ("robot*" OR "social bot*" OR "dialogue system*" OR "conversational agent*" OR "conversational bot*" OR "conversational system*" OR "conversational interface*" OR "chatbot*" OR "chat bot*" OR "chatterbot*" OR "chatter bot*" OR "chat-bot*" OR "smartbot*" OR "smart bot*" OR "smart-bot*" OR "virtual coach*" OR "virtual agent*" OR "embodied agent*" OR "relational agent*" OR "avatar*" OR "virtual character*" OR "animated character*" OR "virtual human*" OR "virtual assistant*" OR "digital assistant*" OR "counseling agent*")).ti,ab.

CINAHL	AB (("mental illness" OR "mental disorder*" OR "affective disorder*" OR "psychotic disorder*" OR "post-traumatic stress disorder*" OR "PTSD" OR distress OR "depress*" OR anxiety OR bipolar OR schizophrenia OR psychosis OR "mental health" OR "mental wellness" OR wellbeing OR "well-being" OR "SWB" OR happiness OR happy OR "positive affect*" OR "negative affect*" OR "positive emotion*" OR "negative emotion*" OR mood OR "life satisfaction" OR "satisfaction with life") AND ("robot*" OR "social bot*" OR "dialogue system*" OR "conversational agent*" OR "conversational bot*" OR "conversational system*" OR "conversational interface*" OR "chatbot*" OR "chat bot*" OR "chatterbot*" OR "chatter bot*" OR "chat-bot*" OR "smartbot*" OR "smart bot*" OR "smart-bot*" OR "virtual coach*" OR "virtual agent*" OR "embodied agent*" OR "relational agent*" OR "avatar*" OR "virtual character*" OR "animated character*" OR "virtual human*" OR "virtual assistant*" OR "digital assistant*" OR "counseling agent*")) OR TI (("mental illness" OR "mental disorder*" OR "affective disorder*" OR "psychotic disorder*" OR "post-traumatic stress disorder*" OR "PTSD" OR distress OR "depress*" OR anxiety OR bipolar OR schizophrenia OR psychosis OR "mental health" OR "mental wellness" OR wellbeing OR "well-being" OR "SWB" OR happiness OR happy OR "positive affect*" OR "negative affect*" OR "positive emotion*" OR "negative emotion*" OR mood OR "life satisfaction" OR "satisfaction with life") AND ("robot*" OR "social bot*" OR "dialogue system*" OR "conversational agent*" OR "conversational bot*" OR "conversational system*" OR "conversational interface*" OR "chatbot*" OR "chat bot*" OR "chatterbot*" OR "chatter bot*" OR "chat-bot*" OR "smartbot*" OR "smart bot*" OR "smart-bot*" OR "virtual coach*" OR "virtual agent*" OR "embodied agent*" OR "relational agent*" OR "avatar*" OR "virtual character*" OR "animated character*" OR "virtual human*" OR "virtual assistant*" OR "digital assistant*" OR "counseling agent*"))
PsycInfo	AB (("mental illness" OR "mental disorder*" OR "affective disorder*" OR "psychotic disorder*" OR "post-traumatic stress disorder*" OR "PTSD" OR distress OR "depress*" OR anxiety OR bipolar OR schizophrenia OR psychosis OR "mental health" OR "mental wellness" OR wellbeing OR "well-being" OR "SWB" OR happiness OR happy OR "positive affect*" OR "negative affect*" OR "positive emotion*" OR "negative emotion*" OR mood OR "life satisfaction" OR "satisfaction with life") AND ("robot*" OR "social bot*" OR "dialogue system*" OR "conversational agent*" OR "conversational bot*" OR "conversational system*" OR "conversational interface*" OR "chatbot*" OR "chat bot*" OR "chatterbot*" OR "chatter bot*" OR "chat-bot*" OR "smartbot*" OR "smart bot*" OR "smart-bot*" OR "virtual coach*" OR "virtual agent*" OR "embodied agent*" OR "relational agent*" OR "avatar*" OR "virtual character*" OR "animated character*" OR "virtual human*" OR "virtual assistant*" OR "digital assistant*" OR "counseling agent*")) OR TI (("mental illness" OR "mental disorder*" OR "affective disorder*" OR "psychotic disorder*" OR "post-traumatic stress disorder*" OR "PTSD" OR distress OR "depress*" OR anxiety OR bipolar OR schizophrenia OR psychosis OR "mental health" OR "mental wellness" OR wellbeing OR "well-being" OR "SWB" OR happiness OR happy OR "positive affect*" OR "negative affect*" OR "positive emotion*" OR "negative emotion*" OR mood OR "life satisfaction" OR "satisfaction with life") AND ("robot*" OR "social bot*" OR "dialogue system*" OR

	"conversational agent*" OR "conversational bot*" OR "conversational system*" OR "conversational interface*" OR "chatbot*" OR "chat bot*" OR "chatterbot*" OR "chatter bot*" OR "chat-bot*" OR "smartbot*" OR "smart bot*" OR "smart-bot*" OR "virtual coach*" OR "virtual agent*" OR "embodied agent*" OR "relational agent*" OR "avatar*" OR "virtual character*" OR "animated character*" OR "virtual human*" OR "virtual assistant*" OR "digital assistant*" OR "counseling agent*"))
Web of Science Core Collection	TI=(("mental illness" OR "mental disorder*" OR "affective disorder*" OR "psychotic disorder*" OR "post-traumatic stress disorder*" OR "PTSD" OR distress OR "depress*" OR anxiety OR bipolar OR schizophrenia OR psychosis OR "mental health" OR "mental wellness" OR wellbeing OR "well-being" OR "SWB" OR happiness OR happy OR "positive affect*" OR "negative affect*" OR "positive emotion*" OR "negative emotion*" OR mood OR "life satisfaction" OR "satisfaction with life") AND ("robot*" OR "social bot*" OR "dialogue system*" OR "conversational agent*" OR "conversational bot*" OR "conversational system*" OR "conversational interface*" OR "chatbot*" OR "chat bot*" OR "chatterbot*" OR "chatter bot*" OR "chat-bot*" OR "smartbot*" OR "smart bot*" OR "smart-bot*" OR "virtual coach*" OR "virtual agent*" OR "embodied agent*" OR "relational agent*" OR "avatar*" OR "virtual character*" OR "animated character*" OR "virtual human*" OR "virtual assistant*" OR "digital assistant*" OR "counseling agent*")) OR AB=(("mental illness" OR "mental disorder*" OR "affective disorder*" OR "psychotic disorder*" OR "post-traumatic stress disorder*" OR "PTSD" OR distress OR "depress*" OR anxiety OR bipolar OR schizophrenia OR psychosis OR "mental health" OR "mental wellness" OR wellbeing OR "well-being" OR "SWB" OR happiness OR happy OR "positive affect*" OR "negative affect*" OR "positive emotion*" OR "negative emotion*" OR mood OR "life satisfaction" OR "satisfaction with life") AND ("robot*" OR "social bot*" OR "dialogue system*" OR "conversational agent*" OR "conversational bot*" OR "conversational system*" OR "conversational interface*" OR "chatbot*" OR "chat bot*" OR "chatterbot*" OR "chatter bot*" OR "chat-bot*" OR "smartbot*" OR "smart bot*" OR "smart-bot*" OR "virtual coach*" OR "virtual agent*" OR "embodied agent*" OR "relational agent*" OR "avatar*" OR "virtual character*" OR "animated character*" OR "virtual human*" OR "virtual assistant*" OR "digital assistant*" OR "counseling agent*"))

EBSCO-Communication Source	AB (("mental illness" OR "mental disorder*" OR "affective disorder*" OR "psychotic disorder*" OR "post-traumatic stress disorder*" OR "PTSD" OR distress OR "depress*" OR anxiety OR bipolar OR schizophrenia OR psychosis OR "mental health" OR "mental wellness" OR wellbeing OR "well-being" OR "SWB" OR happiness OR happy OR "positive affect*" OR "negative affect*" OR "positive emotion*" OR "negative emotion*" OR mood OR "life satisfaction" OR "satisfaction with life") AND ("robot*" OR "social bot*" OR "dialogue system*" OR "conversational agent*" OR "conversational bot*" OR "conversational system*" OR "conversational interface*" OR "chatbot*" OR "chat bot*" OR "chatterbot*" OR "chatter bot*" OR "chat-bot*" OR "smartbot*" OR "smart bot*" OR "smart-bot*" OR "virtual coach*" OR "virtual agent*" OR "embodied agent*" OR "relational agent*" OR "avatar*" OR "virtual character*" OR "animated character*" OR "virtual human*" OR "virtual assistant*" OR "digital assistant*" OR "counseling agent*")) OR TI (("mental illness" OR "mental disorder*" OR "affective disorder*" OR "psychotic disorder*" OR "post-traumatic stress disorder*" OR "PTSD" OR distress OR "depress*" OR anxiety OR bipolar OR schizophrenia OR psychosis OR "mental health" OR "mental wellness" OR wellbeing OR "well-being" OR "SWB" OR happiness OR happy OR "positive affect*" OR "negative affect*" OR "positive emotion*" OR "negative emotion*" OR mood OR "life satisfaction" OR "satisfaction with life") AND ("robot*" OR "social bot*" OR "dialogue system*" OR "conversational agent*" OR "conversational bot*" OR "conversational system*" OR "conversational interface*" OR "chatbot*" OR "chat bot*" OR "chatterbot*" OR "chatter bot*" OR "chat-bot*" OR "smartbot*" OR "smart bot*" OR "smart-bot*" OR "virtual coach*" OR "virtual agent*" OR "embodied agent*" OR "relational agent*" OR "avatar*" OR "virtual character*" OR "animated character*" OR "virtual human*" OR "virtual assistant*" OR "digital assistant*" OR "counseling agent*"))
ProQuest Dissertations & Theses Global	ab(("mental illness" OR "mental disorder*" OR "affective disorder*" OR "psychotic disorder*" OR "post-traumatic stress disorder*" OR "PTSD" OR distress OR "depress*" OR anxiety OR bipolar OR schizophrenia OR psychosis OR "mental health" OR "mental wellness" OR wellbeing OR "well-being" OR "SWB" OR happiness OR happy OR "positive affect*" OR "negative affect*" OR "positive emotion*" OR "negative emotion*" OR mood OR "life satisfaction" OR "satisfaction with life") AND ("robot*" OR "social bot*" OR "dialogue system*" OR "conversational agent*" OR "conversational bot*" OR "conversational system*" OR "conversational interface*" OR "chatbot*" OR "chat bot*" OR "chatterbot*" OR "chatter bot*" OR "chat-bot*" OR "smartbot*" OR "smart bot*" OR "smart-bot*" OR "virtual coach*" OR "virtual agent*" OR "embodied agent*" OR "relational agent*" OR "avatar*" OR "virtual character*" OR "animated character*" OR "virtual human*" OR "virtual assistant*" OR "digital assistant*" OR "counseling agent*")) OR ti(("mental illness" OR "mental disorder*" OR "affective disorder*" OR "psychotic disorder*" OR "post-traumatic stress disorder*" OR "PTSD" OR distress OR "depress*" OR anxiety OR bipolar OR schizophrenia OR psychosis OR "mental health" OR "mental wellness" OR wellbeing OR "well-being" OR "SWB" OR happiness OR happy OR "positive affect*" OR "negative affect*" OR "positive emotion*" OR "negative emotion*" OR mood OR "life satisfaction" OR "satisfaction with life") AND ("robot*" OR "social bot*" OR "dialogue system*" OR

	"conversational agent*" OR "conversational bot*" OR "conversational system*" OR "conversational interface*" OR "chatbot*" OR "chat bot*" OR "chatterbot*" OR "chatter bot*" OR "chat-bot*" OR "smartbot*" OR "smart bot*" OR "smart-bot*" OR "virtual coach*" OR "virtual agent*" OR "embodied agent*" OR "relational agent*" OR "avatar*" OR "virtual character*" OR "animated character*" OR "virtual human*" OR "virtual assistant*" OR "digital assistant*" OR "counseling agent*"))
OSF Preprints	title:(+("mental illness" OR "mental disorder*" OR "affective disorder*" OR "psychotic disorder*" OR "post\-traumatic stress disorder*" OR "PTSD" OR distress OR "depress*" OR anxiety OR bipolar OR schizophrenia OR psychosis OR "mental health" OR "mental wellness" OR wellbeing OR "well\-being" OR "SWB" OR happiness OR happy OR "positive affect*" OR "negative affect*" OR "positive emotion*" OR "negative emotion*" OR mood OR "life satisfaction" OR "satisfaction with life") +("robot*" OR "social bot*" OR "dialogue system*" OR "conversational agent*" OR "conversational bot*" OR "conversational system*" OR "conversational interface*" OR "chatbot*" OR "chat bot*" OR "chatterbot*" OR "chatter bot*" OR "chat\-bot*" OR "smartbot*" OR "smart bot*" OR "smart\-bot*" OR "virtual coach*" OR "virtual agent*" OR "embodied agent*" OR "relational agent*" OR "avatar*" OR "virtual character*" OR "animated character*" OR "virtual human*" OR "virtual assistant*" OR "digital assistant*" OR "counseling agent*"))
PsyArXiv	title-abstract: (("mental illness" OR "mental disorder*" OR "affective disorder*" OR "psychotic disorder*" OR "post-traumatic stress disorder*" OR "PTSD" OR distress OR "depress*" OR anxiety OR bipolar OR schizophrenia OR psychosis OR "mental health" OR "mental wellness" OR wellbeing OR "well-being" OR "SWB" OR happiness OR happy OR "positive affect*" OR "negative affect*" OR "positive emotion*" OR "negative emotion*" OR mood OR "life satisfaction" OR "satisfaction with life")) AND (("robot*" OR "social bot*" OR "dialogue system*" OR "conversational agent*" OR "conversational bot*" OR "conversational system*" OR "conversational interface*" OR "chatbot*" OR "chat bot*" OR "chatterbot*" OR "chatter bot*" OR "chat-bot*" OR "smartbot*" OR "smart bot*" OR "smart-bot*" OR "virtual coach*" OR "virtual agent*" OR "embodied agent*" OR "relational agent*" OR "avatar*" OR "virtual character*" OR "animated character*" OR "virtual human*" OR "virtual assistant*" OR "digital assistant*" OR "counseling agent*"))

EuropePMC	(((TITLE:"mental illness" OR TITLE:"mental disorder*" OR TITLE:"affective disorder*" OR TITLE:"psychotic disorder*" OR TITLE:"post-traumatic stress disorder*" OR TITLE:"PTSD" OR TITLE:distress OR TITLE:"depress*" OR TITLE:anxiety OR TITLE:bipolar OR TITLE:schizophrenia OR TITLE:psychosis OR TITLE:"mental health" OR TITLE:"mental wellness" OR TITLE:wellbeing OR TITLE:"well-being" OR TITLE:"SWB" OR TITLE:happiness OR TITLE:happy OR TITLE:"positive affect*" OR TITLE:"negative affect*" OR TITLE:"positive emotion*" OR TITLE:"negative emotion*" OR TITLE:mood OR TITLE:"life satisfaction" OR TITLE:"satisfaction with life") AND (TITLE:"robot*" OR TITLE:"social bot*" OR TITLE:"dialogue system*" OR TITLE:"conversational agent*" OR TITLE:"conversational bot*" OR TITLE:"conversational system*" OR TITLE:"conversational interface*" OR TITLE:"chatbot*" OR TITLE:"chat bot*" OR TITLE:"chatterbot*" OR TITLE:"chatter bot*" OR TITLE:"chat-bot*" OR TITLE:"smartbot*" OR TITLE:"smart bot*" OR TITLE:"smart-bot*" OR TITLE:"virtual coach*" OR TITLE:"virtual agent*" OR TITLE:"embodied agent*" OR TITLE:"relational agent*" OR TITLE:"avatar*" OR TITLE:"virtual character*" OR TITLE:"animated character*" OR TITLE:"virtual human*" OR TITLE:"virtual assistant*" OR TITLE:"digital assistant*" OR TITLE:"counseling agent*"))) OR (((ABSTRACT:"mental illness" OR ABSTRACT:"mental disorder*" OR ABSTRACT:"affective disorder*" OR ABSTRACT:"psychotic disorder*" OR ABSTRACT:"post-traumatic stress disorder*" OR ABSTRACT:"PTSD" OR ABSTRACT:distress OR ABSTRACT:"depress*" OR ABSTRACT:anxiety OR ABSTRACT:bipolar OR ABSTRACT:schizophrenia OR ABSTRACT:psychosis OR ABSTRACT:"mental health" OR ABSTRACT:"mental wellness" OR ABSTRACT:wellbeing OR ABSTRACT:"well-being" OR ABSTRACT:"SWB" OR ABSTRACT:happiness OR ABSTRACT:happy OR ABSTRACT:"positive affect*" OR ABSTRACT:"negative affect*" OR ABSTRACT:"positive emotion*" OR ABSTRACT:"negative emotion*" OR ABSTRACT:mood OR ABSTRACT:"life satisfaction" OR ABSTRACT:"satisfaction with life") AND (ABSTRACT:"robot*" OR ABSTRACT:"social bot*" OR ABSTRACT:"dialogue system*" OR ABSTRACT:"conversational agent*" OR ABSTRACT:"conversational bot*" OR ABSTRACT:"conversational system*" OR ABSTRACT:"conversational interface*" OR ABSTRACT:"chatbot*" OR ABSTRACT:"chat bot*" OR ABSTRACT:"chatterbot*" OR ABSTRACT:"chatter bot*" OR ABSTRACT:"chat-bot*" OR ABSTRACT:"smartbot*" OR ABSTRACT:"smart bot*" OR ABSTRACT:"smart-bot*" OR ABSTRACT:"virtual coach*" OR ABSTRACT:"virtual agent*" OR ABSTRACT:"embodied agent*" OR ABSTRACT:"relational agent*" OR ABSTRACT:"avatar*" OR ABSTRACT:"virtual character*" OR ABSTRACT:"animated character*" OR ABSTRACT:"virtual human*" OR ABSTRACT:"virtual assistant*" OR ABSTRACT:"digital assistant*" OR ABSTRACT:"counseling agent*"))) AND (((SRC:MED OR SRC:PMC OR SRC:AGR OR SRC:CBA) NOT (PUB_TYPE:"Review")) OR SRC:PPR)

Supplementary Table 8 Eligibility criteria for study inclusion

Criteria	Inclusion criteria	Exclusion criteria
Use of an AI-based conversational agent	We defined AI-based conversational agents (CAs) as software agents or bots that leverage NLP, machine learning or other AI models and techniques to simulate human-like conversations. These agents possess the capability to understand user intent, analyze contexts, and retrieve or generate appropriate responses, in contrast to their rule-based counterparts, which solely rely on predefined rules or decision trees to formulate responses. The CA can take the form of either text or voice-based and can be embodied or non-embodied. It can function as a stand-alone system, work via a web browser, or be integrated into messaging applications. CAs that are part of virtual reality, augmented reality and robots were also included.	The hardware configuration of the CA was not a criterion for exclusion. However, the inclusion criteria necessitated the use of an AI-based CA. For example, rule-based CAs that formulate responses to user queries through a predetermined set of rules without employing any AI algorithms or techniques were excluded (e.g.,[35]). Moreover, studies employing CAs that limited user input to predetermined options, such as clicking or tapping on predefined words, phrases, or visuals, and did not allow free-flowing text or voice input were also excluded for consideration.
Involve two-way interactions between the user and the CA	We included studies that collected data from actual users who had engaged in a two-way interaction with the CA as reported in the study. We defined two-way interactions as scenarios in which both the CA and the user are actively engaged.	Studies that solely focused on the design, algorithm, architecture, and development of CAs, without any empirical evaluation of the interaction between the user and the CA (e.g.,[36]) were excluded from our review. Additionally, we excluded studies that only involved one-way instructions conveyed by the CA to users without any responses from the users.
Include self-report and/or objective measures of mental distress or mental well-being and link these measures to CA use.	To ensure inclusivity, a broad range of mental health outcomes were considered eligible, ranging from validated psychiatric rating scales (e.g., PHQ-9), through non-validated custom rating scales, to objective measures derived from passive sensing systems (e.g., unobtrusively collected audio or visual biomarkers) or third-party evaluations. Mental distress measures encompass the common mental disorders identified by the WHO[37], including depression, anxiety disorder, post-traumatic stress disorder, bipolar disorder, and schizophrenia, as well as other distress measures such as pain, fatigue, stress, and general distress. Beyond the traditional focus on mental disorders, we also included	Studies were excluded if they lacked any measures of mental disorder, distress or mental well-being. This included studies solely focused on non-clinical user perception or experiences (e.g.,[38]) or those that only reported qualitative accounts of changes in mental health attributed to the use of AI-based CAs (e.g.,[39]).

	positive aspects of mental health. Mental well-being measures included both short-term emotional states and moods, as well as long-term measures such as life satisfaction.	

Supplementary Table 9 PRISMA Checklists

Section and Topic	Item #	Checklist item	Location where item is reported #page number
**TITLE**			
Title	1	Identify the report as a systematic review.	1
**ABSTRACT**			
Abstract	2	See the PRISMA 2020 for Abstracts checklist.	2
**INTRODUCTION**			
Rationale	3	Describe the rationale for the review in the context of existing knowledge.	3-4
Objectives	4	Provide an explicit statement of the objective(s) or question(s) the review addresses.	4
**METHODS**			
Eligibility criteria	5	Specify the inclusion and exclusion criteria for the review and how studies were grouped for the syntheses.	17; Supplementary Table 8
Information sources	6	Specify all databases, registers, websites, organisations, reference lists and other sources searched or consulted to identify studies. Specify the date when each source was last searched or consulted.	16; Supplementary Table 7
Search strategy	7	Present the full search strategies for all databases, registers and websites, including any filters and limits used.	16; Supplementary Table 7
Selection process	8	Specify the methods used to decide whether a study met the inclusion criteria of the review, including how many reviewers screened each record and each report retrieved, whether they worked independently, and if applicable, details of automation tools used in the process.	16-17
Data collection process	9	Specify the methods used to collect data from reports, including how many reviewers collected data from each report, whether they worked independently, any processes for obtaining or confirming data from study investigators, and if applicable, details of automation tools used in the process.	17-18
Data items	10a	List and define all outcomes for which data were sought. Specify whether all results that were compatible with each outcome domain in each study were sought (e.g. for all measures, time points, analyses), and if not, the methods used to decide which results to collect.	19
	10b	List and define all other variables for which data were sought (e.g. participant and intervention characteristics, funding sources). Describe any assumptions made about any missing or unclear information.	18
Study risk of bias assessment	11	Specify the methods used to assess risk of bias in the included studies, including details of the tool(s) used, how many reviewers assessed each study and whether they worked independently, and if applicable, details of automation tools used in the process.	21
Effect measures	12	Specify for each outcome the effect measure(s) (e.g. risk ratio, mean difference) used in the synthesis or presentation of results.	19
Synthesis methods	13a	Describe the processes used to decide which studies were eligible for each synthesis (e.g. tabulating the study intervention characteristics and comparing against the planned groups for each synthesis (item #5)).	18-19

Section and Topic	Item #	Checklist item	Location where item is reported #page number
	13b	Describe any methods required to prepare the data for presentation or synthesis, such as handling of missing summary statistics, or data conversions.	18
	13c	Describe any methods used to tabulate or visually display results of individual studies and syntheses.	18
	13d	Describe any methods used to synthesize results and provide a rationale for the choice(s). If meta-analysis was performed, describe the model(s), method(s) to identify the presence and extent of statistical heterogeneity, and software package(s) used.	19
	13e	Describe any methods used to explore possible causes of heterogeneity among study results (e.g. subgroup analysis, meta-regression).	20
	13f	Describe any sensitivity analyses conducted to assess robustness of the synthesized results.	20
Reporting bias assessment	14	Describe any methods used to assess risk of bias due to missing results in a synthesis (arising from reporting biases).	21
Certainty assessment	15	Describe any methods used to assess certainty (or confidence) in the body of evidence for an outcome.	21-22
**RESULTS**			
Study selection	16a	Describe the results of the search and selection process, from the number of records identified in the search to the number of studies included in the review, ideally using a flow diagram.	5
	16b	Cite studies that might appear to meet the inclusion criteria, but which were excluded, and explain why they were excluded.	Supplementary Table 8
Study characteristics	17	Cite each included study and present its characteristics.	5; Supplementary Table 1 &Table 2
Risk of bias in studies	18	Present assessments of risk of bias for each included study.	10, Supplementary Fig. 5
Results of individual studies	19	For all outcomes, present, for each study: (a) summary statistics for each group (where appropriate) and (b) an effect estimate and its precision (e.g. confidence/credible interval), ideally using structured tables or plots.	9; Supplementary Fig. 1, Fig. 2, Fig. 3, Fig. 4
Results of syntheses	20a	For each synthesis, briefly summarise the characteristics and risk of bias among contributing studies.	10
	20b	Present results of all statistical syntheses conducted. If meta-analysis was done, present for each the summary estimate and its precision (e.g. confidence/credible interval) and measures of statistical heterogeneity. If comparing groups, describe the direction of the effect.	9; Supplementary Fig. 1, Fig. 2, Fig. 3, Fig. 4
	20c	Present results of all investigations of possible causes of heterogeneity among study results.	11
	20d	Present results of all sensitivity analyses conducted to assess the robustness of the synthesized results.	9; Supplementary Table 3
Reporting biases	21	Present assessments of risk of bias due to missing results (arising from reporting biases) for each synthesis assessed.	10; Supplementary Table 4

Section and Topic	Item #	Checklist item	Location where item is reported #page number
Certainty of evidence	22	Present assessments of certainty (or confidence) in the body of evidence for each outcome assessed.	10; Supplementary Table 5
**DISCUSSION**			
Discussion	23a	Provide a general interpretation of the results in the context of other evidence.	12-15
	23b	Discuss any limitations of the evidence included in the review.	12-15
	23c	Discuss any limitations of the review processes used.	15-16
	23d	Discuss implications of the results for practice, policy, and future research.	15-16
**OTHER INFORMATION**			
Registration and protocol	24a	Provide registration information for the review, including register name and registration number, or state that the review was not registered.	22
	24b	Indicate where the review protocol can be accessed, or state that a protocol was not prepared.	22
	24c	Describe and explain any amendments to information provided at registration or in the protocol.	N/A
Support	25	Describe sources of financial or non-financial support for the review, and the role of the funders or sponsors in the review.	22
Competing interests	26	Declare any competing interests of review authors.	22
Availability of data, code and other materials	27	Report which of the following are publicly available and where they can be found: template data collection forms; data extracted from included studies; data used for all analyses; analytic code; any other materials used in the review.	22

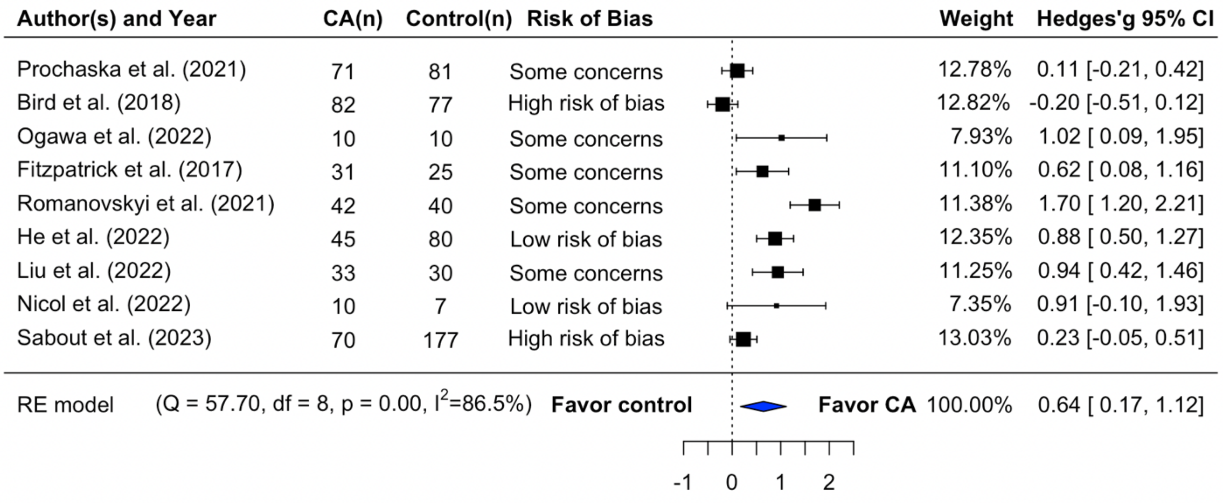

Supplementary Fig 1. Effects of CA interventions on depressive symptoms

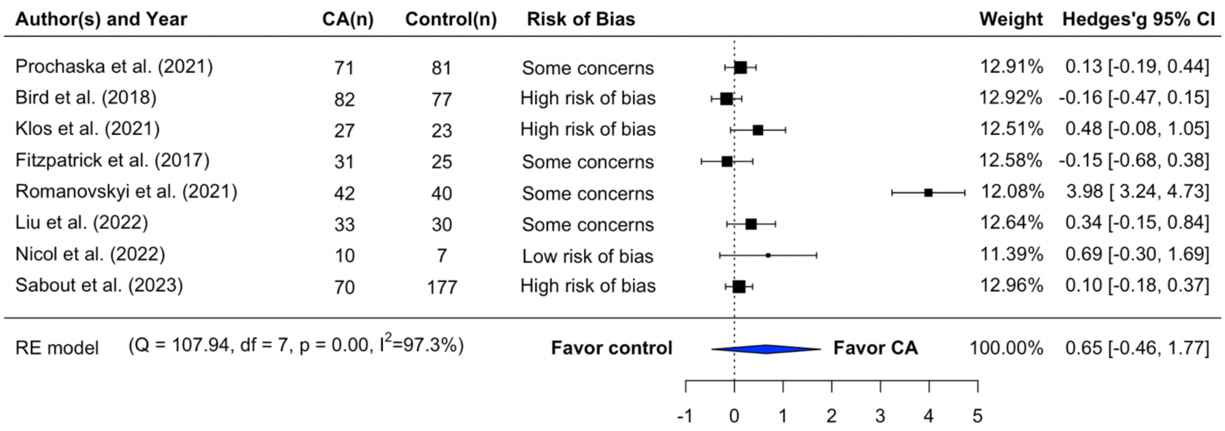

Supplementary Fig 2. Effects of CA interventions on generalized anxiety symptoms

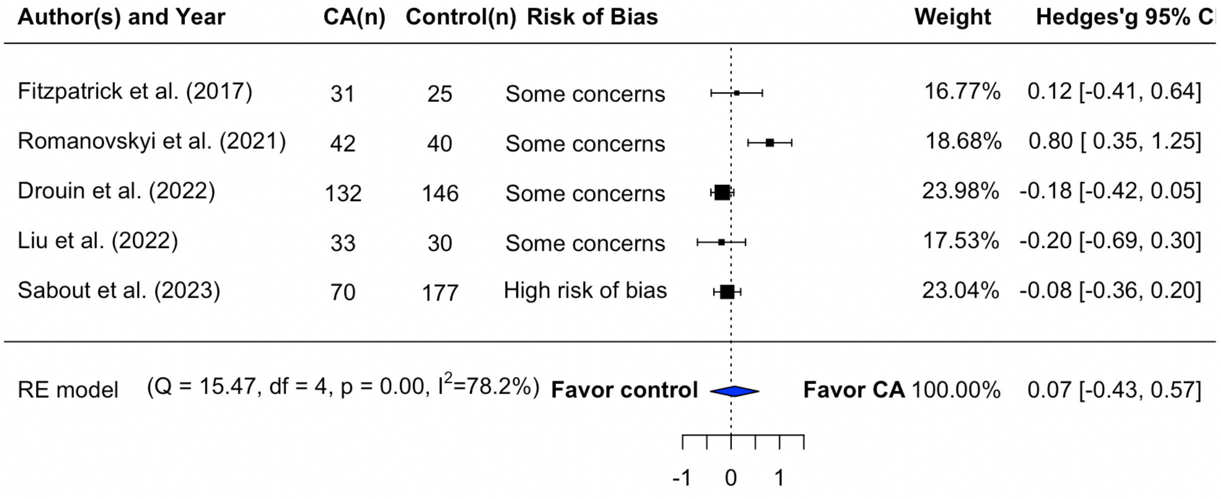

Supplementary Fig 3. Effects of CA interventions on positive affect

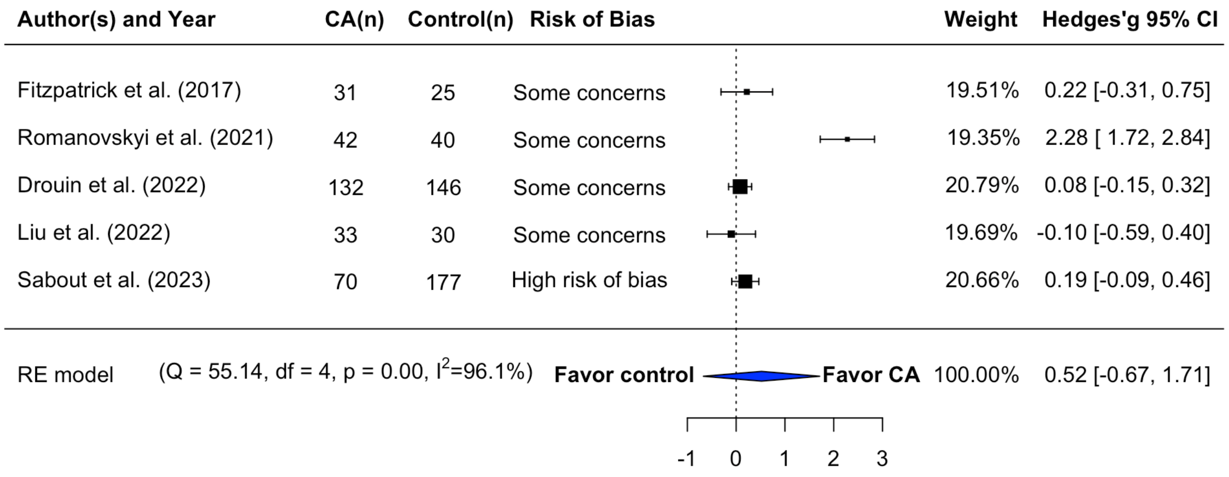

Supplementary Fig 4. Effects of CA interventions on negative affect

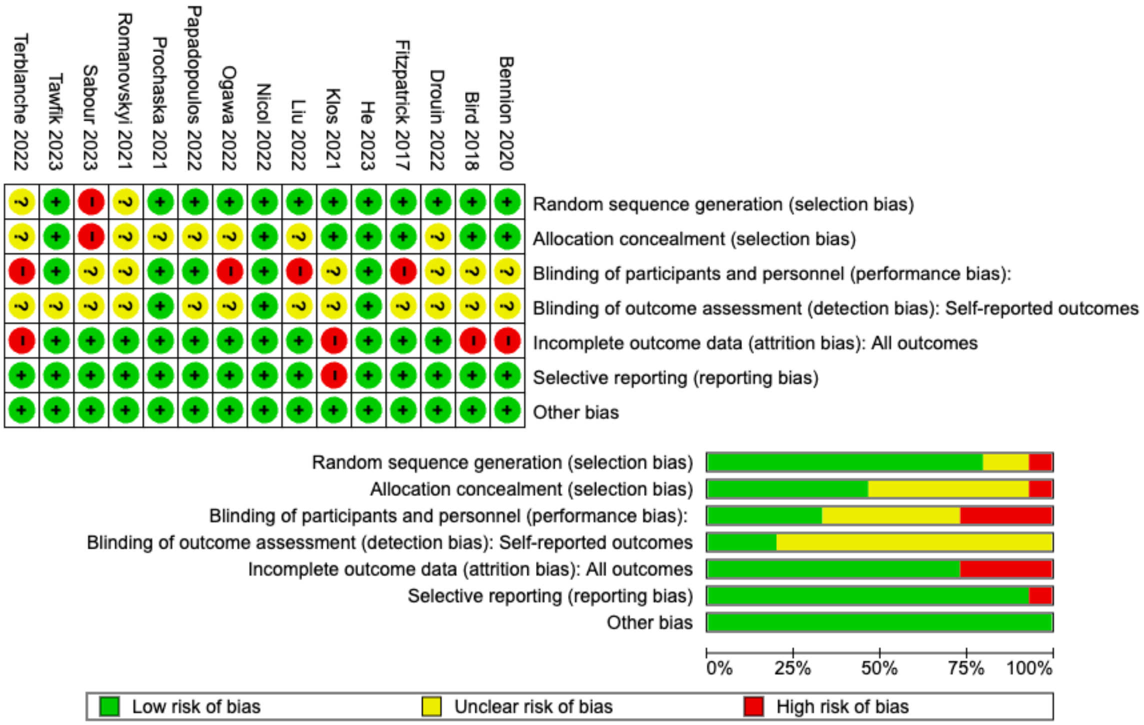

Supplementary Fig 5. Risk of bias of RCTs

References

1. Prochaska, J. J. *et al.* A randomized controlled trial of a therapeutic relational agent for reducing substance misuse during the COVID-19 pandemic. *Drug Alcohol Depend.* **227**, 108986 (2021).
2. Bird, T., Mansell, W., Wright, J., Gaffney, H. & Tai, S. Manage your life online: A web-based randomized controlled trial evaluating the effectiveness of a problem-solving intervention in a student sample. *Behav. Cogn. Psychother.* **46**, 570–582 (2018).
3. Klos, M. C. *et al.* Artificial Intelligence–based chatbot for anxiety and depression in university students: Pilot randomized controlled trial. *JMIR Formative Research* **5**, e20678 (2021).
4. Ogawa, M. *et al.* Can AI make people happy? The effect of AI-based chatbot on smile and speech in Parkinson's disease. *Parkinsonism Relat. Disord.* **99**, 43–46 (2022).
5. Terblanche, N., Molyn, J., De Haan, E. & Nilsson, V. O. Coaching at scale: Investigating the efficacy of Artificial Intelligence coaching. *International Journal of Evidence Based Coaching and Mentoring* (2022).
6. Fitzpatrick, K. K., Darcy, A. & Vierhile, M. Delivering cognitive behavior therapy to young adults with symptoms of depression and anxiety using a fully automated conversational agent (Woebot): A randomized controlled trial. *JMIR Ment Health* **4**, e19 (2017).
7. Romanovskyi, O., Pidbutska, N. & Knysh, A. Elomia Chatbot: The effectiveness of Artificial Intelligence in the fight for mental health. in *COLINS* 1215–1224 (2021).
8. Drouin, M., Sprecher, S., Nicola, R. & Perkins, T. Is chatting with a sophisticated chatbot as good as chatting online or FTF with a stranger? *Comput. Human Behav.* **128**, 107100 (2022).

9. He, Y. *et al.* Mental health chatbot for young adults with depressive symptoms during the COVID-19 pandemic: Single-blind, three-arm randomized controlled trial. *J. Med. Internet Res.* **24**, e40719 (2022).
10. Papadopoulos, C. *et al.* The CARESSES randomised controlled trial: Exploring the health-related impact of culturally competent Artificial Intelligence embedded into socially assistive robots and tested in older adult care homes. *Adv. Robot.* **14**, 245–256 (2022).
11. Bennion, M. R., Hardy, G. E., Moore, R. K., Kellett, S. & Millings, A. Usability, acceptability, and effectiveness of web-based conversational agents to facilitate problem solving in older adults: Controlled study. *J. Med. Internet Res.* **22**, e16794 (2020).
12. Liu, H., Peng, H., Song, X., Xu, C. & Zhang, M. Using AI chatbots to provide self-help depression interventions for university students: A randomized trial of effectiveness. *Internet Interv* **27**, 100495 (2022).
13. Nicol, G., Wang, R., Graham, S., Dodd, S. & Garbutt, J. Chatbot-delivered cognitive behavioral therapy in adolescents with depression and anxiety during the COVID-19 pandemic: Feasibility and acceptability study. *JMIR Form Res* **6**, e40242 (2022).
14. Tawfik, E., Ghallab, E. & Moustafa, A. A nurse versus a chatbot – the effect of an empowerment program on chemotherapy-related side effects and the self-care behaviors of women living with breast Cancer: a randomized controlled trial. *BMC Nurs.* **22**, 102 (2023).
15. Sabour, S. et al. Chatbots for mental health support: Exploring the impact of Emohaa on reducing mental distress in China. *arXiv [cs.CL]* (2022).
16. Fulmer, R., Joerin, A., Gentile, B., Lakerink, L. & Rauws, M. Using psychological Artificial Intelligence (Tess) to relieve symptoms of depression and anxiety: Randomized controlled trial. *JMIR Ment Health* **5**, e64 (2018).

17. Vertsberger, D., Winsberg, M. & Naor, N. Adolescents' wellbeing while using a mobile AI-powered acceptance commitment therapy tool: Evidence from a longitudinal study. *PsyArXiv* (2022).

18. Leo, A. J. *et al.* A digital mental health intervention in an orthopedic setting for patients with symptoms of depression and/or anxiety: Feasibility prospective cohort study. *JMIR Form Res* **6**, e34889 (2022).

19. Rathnayaka, P. *et al.* A mental health chatbot with cognitive skills for personalised behavioural activation and remote health monitoring. *Sensors* **22**, (2022).

20. Abdollahi, H., Mollahosseini, A., Lane, J. T. & Mahoor, M. H. A pilot study on using an intelligent life-like robot as a companion for elderly individuals with dementia and depression. in *2017 IEEE-RAS 17th International Conference on Humanoid Robotics (Humanoids)* 541–546 (2017).

21. Prochaska, J. J. *et al.* A therapeutic relational agent for reducing problematic substance use (Woebot): Development and usability study. *J. Med. Internet Res.* **23**, e24850 (2021).

22. Bassi, G. *et al.* A virtual coach (Motibot) for supporting healthy coping strategies among adults with diabetes: Proof-of-Concept study. *JMIR Hum Factors* **9**, e32211 (2022).

23. Goga, N. *et al.* An efficient system for Eye Movement Desensitization and Reprocessing (EMDR) therapy: A pilot study. *Healthcare (Basel)* **10**, (2022).

24. Tulsulkar, G. *et al.* Can a humanoid social robot stimulate the interactivity of cognitively impaired elderly? A thorough study based on computer vision methods. *Vis. Comput.* **37**, 3019–3038 (2021).

25. Trappey, A. J. C., Lin, A. P. C., Hsu, K. Y. K., Trappey, C. V. & Tu, K. L. K. Development

of an empathy-centric counseling chatbot system capable of sentimental dialogue analysis. *Processes* **10**, 930 (2022).

26. Leo, A. J. *et al.* Digital mental health intervention plus usual care compared with usual care only and usual care plus in-person psychological counseling for orthopedic patients with symptoms of depression or anxiety: Cohort study. *JMIR Formative Research* **6**, e36203 (2022).
27. De Nieva, J. O., Joaquin, J. A., Tan, C. B., Marc Te, R. K. & Ong, E. Investigating students' use of a mental health chatbot to alleviate academic stress. in *6th International ACM In-Cooperation HCI and UX Conference* (ACM, 2020).
28. Gamborino, E., Yueh, H.-P., Lin, W., Yeh, S.-L. & Fu, L.-C. Mood estimation as a social profile predictor in an autonomous, multi-session, emotional support robot for children. in *2019 28th IEEE International Conference on Robot and Human Interactive Communication (RO-MAN)* 1–6 (2019).
29. Pham, M., Do, H. M., Su, Z., Bishop, A. & Sheng, W. Negative emotion management using a smart shirt and a robot assistant. *IEEE Robotics and Automation Letters* **6**, 4040–4047 (2021).
30. Daley, K. *et al.* Preliminary evaluation of the engagement and effectiveness of a mental health chatbot. *Front Digit Health* **2**, 576361 (2020).
31. Demirci, H. M. User experience over time with conversational agents: Case study of woebot on supporting subjective well-being. (Middle East Technical University, 2018).
32. Legaspi, C. M., Jr, Pacana, T. R., Loja, K., Sing, C. & Ong, E. User perception of Wysa as a mental well-being support tool during the COVID-19 pandemic. in *Asian HCI Symposium'22* 52–57 (Association for Computing Machinery, 2023).

33. Wrightson-Hester, A.-R. et al. Manage Your Life Online ('MYLO'): Co-design and case-series of an artificial therapist to support youth mental health. *PsyArXiv* (2023).

34. Chiauzzi, E. et al. Demographic and clinical characteristics associated with anxiety and depressive symptom outcomes in users of a digital mental health intervention incorporating a relational agent. *Research Square* (2023).

35. Ly KH, Ly A-M, Andersson G. A fully automated conversational agent for promoting mental well-being: A pilot RCT using mixed methods. *Internet Interv*. **10**: 39–46 (2017).

36. Saha T, Gakhreja V, Das AS, Chakraborty S, Saha S. Towards Motivational and Empathetic Response Generation in Online Mental Health Support. In *Proceedings of the 45th International ACM SIGIR Conference on Research and Development in Information Retrieval* 2650-6 (Association for Computing Machinery, 2022).

37. Charlson F, van Ommeren M, Flaxman A, Cornett J, Whiteford H, Saxena S. New WHO prevalence estimates of mental disorders in conflict settings: a systematic review and meta-analysis. *Lancet*. **394**: 240–8 (2019).

38. Valagkouti IA, Troussas C, Krouska A, Feidakis M, Sgouropoulou C. Emotion recognition in human–robot interaction using the NAO robot. *Computers*. **11**: 72 (2022).

39. Jiang Q, Zhang Y, Pian W. Chatbot as an emergency exist: Mediated empathy for resilience via human-AI interaction during the COVID-19 pandemic. *Inf Process Manag.* **59**: 103074 (2022).
